# Comparative in Silico Analysis of Ferric Reduction Oxidase (FRO) Genes Expression Patterns in Response to Abiotic Stresses, Metal and Hormone Applications

**DOI:** 10.3390/molecules23051163

**Published:** 2018-05-12

**Authors:** Izhar Muhammad, Xiu-Qing Jing, Abdullah Shalmani, Muhammad Ali, Shi Yi, Peng-Fei Gan, Wen-Qiang Li, Wen-Ting Liu, Kun-Ming Chen

**Affiliations:** 1 State Key Laboratory of Crop Stress Biology in Arid Areas, College of Life Sciences, Northwest A&F University, Yangling 712100, China; izeyaar@gmail.com (I.M.); xiuqingjing@nwafu.edu.cn (X.-Q.J.); abdullqadir36@yahoo.com (A.S.); shiyi1003@nwafu.edu.cn (S.Y.); gpf@nwafu.edu.cn (P.-F.G.); wqli@nwsuaf.edu.cn (W.-Q.L.); lwt001975@163.com (W.-T.L.); 2 College of Horticulture, Northwest A&F University, Yangling 712100, China; alinhorti@nwafu.edu.cn

**Keywords:** FRO, ferric reduction oxidase, evolutionary relationship, gene expression, abiotic and metals stresses

## Abstract

The ferric reduction oxidase (FRO) gene family is involved in various biological processes widely found in plants and may play an essential role in metal homeostasis, tolerance and intricate signaling networks in response to a number of abiotic stresses. Our study describes the identification, characterization and evolutionary relationships of FRO genes families. Here, total 50 FRO genes in Plantae and 15 ‘FRO like’ genes in non-Plantae were retrieved from 16 different species. The entire FRO genes have been divided into seven clades according to close similarity in biological and functional behavior. Three conserved domains were common in FRO genes while in two FROs sub genome have an extra NADPH-Ox domain, separating the function of plant FROs. *OsFRO1* and *OsFRO7* genes were expressed constitutively in rice plant. Real-time RT-PCR analysis demonstrated that the expression of *OsFRO1* was high in flag leaf, and *OsFRO7* gene expression was maximum in leaf blade and flag leaf. Both genes showed vigorous expressions level in response to different abiotic and hormones treatments. Moreover, the expression of both genes was also substantial under heavy metal stresses. *OsFRO1* gene expression was triggered following 6 h under Zn, Pb, Co and Ni treatments, whereas *OsFRO7* gene expression under Fe, Pb and Ni after 12 h, Zn and Cr after 6 h, and Mn and Co after 3 h treatments. These findings suggest the possible involvement of both the genes under abiotic and metal stress and the regulation of phytohormones. Therefore, our current work may provide the foundation for further functional characterization of rice FRO genes family.

## 1. Introduction

The plant growth and development greatly affected by the imbalance supply of mineral nutrition, consequently lower the crop productivity [1]. The most crucial transition micronutrient, iron (Fe), is found almost in all living organism, contributing to the redox centers of proteins which are essential for respiration, biosynthesis of chlorophyll and photosynthesis [2]. The rapid changes in the oxidative state of iron stimulate cellular function, regulation, electron transport and various other metabolic functions [3,4,5,6]. The gene regulation networks may alter the expression level in response to Fe toxicity [7], where the transporters and transcription factors are the key factors which are involved in iron translocation [8,9,10,11]. Therefore, the regulation of genes related to iron stress may help in plant adaptation against adverse conditions [12].

The ferric reduction oxidases (FROs) gene encoding ferric reductase activity is executed by the ferric chelate reductase (FCR) enzyme which is mainly positioned in roots and shoots [13]. The FROs cover in the superfamily of flavocytochrome located in the cellular membrane that transfer electrons from intracellular donors to extracellular acceptors such as iron or molecular oxygen [14]. The major functional domains of FRO genes consist of six membrane-spanning regions, two heme, or ferric reductases-like, transmembrane components, which are a highly conserved core protein throughout the flavocytochromes family and crucial for cell surface ferric reductase activity [15,16]. The flavin adenine dinucleotide (FAD-binding-8) and nicotinamide adenine dinucleotide (NAD-binding-6) domains likely coordinate two intramembranous heme groups leading to superoxide formation and are instrumental for electron transfer [15,17]. The FRO homologs are universally present in a broad range of organisms, including fungi, animals and higher plants [18]. The yeast ferric reductases (FREs), which are considered the isoforms of FROs, are involved in the reduction of iron and copper reported in fungi [19,20]. In animals, seven NADPH oxidase (nicotinamide adenine dinucleotide phosphate-oxidase, *NOX*) genes have been noted, designated as *NOX1~5*, *DUOX1* and *DUOX2* [18,21]. In higher plants, FRO genes have been reported in *Arabidopsis* [22], rice [16], pea [23], and tomato [24]. Thereafter, 50 FRO genes were identified in plants [25]. The plant FROs are expressed in different tissues depending on their locations within cell compartments and are responsible for iron homeostasis, transport and stress response [26]. Ferric-chelate reductase (FRE) was first identified in *Arabidopsis* [14]. More recently the subcellular localization of FRO family proteins were identified, where the authors reported that *FRO2*, *FRO3* and *FRO5* are expressed in roots having a role in iron uptake from the soil [27], and *FRO6*, *FRO7*, and *FRO8* are positioned in shoots, while *FRO1* and *FRO4* gene expression occurs in both roots and leaves, but expression is comparatively low [22,28,29,30,31]. In rice only two ‘FRO-like’ genes (*OsFRO1* and *OsFRO7*) were identified, having unique functional characteristics in Fe uptake and abiotic stresses [16,32]. The expression of *OsFRO1* was noticed in Zn^2−^-, Mn^2−^- and Cu^2−^-deficient rice leaves and later their role in iron homeostasis and bulky biomass under Fe toxic condition was confirmed by [33], The rice *OsFRO1* mutant showed more Fe content in grain when grown in iron-toxic soil [33]. Likewise, the knockdown of *LeFRO1* transgene showed its effect on Fe partition in *Nicotiana benthamiana* [26]. The iron deficiency responses necessitate the ethylene signaling pathway, transcription factor, and up-regulation of several genes in *Arabidopsis* [34,35]. Moreover, the auxin-mediated interaction between the ethylene insensitive 3 (*EIN3*) and *FIT* protein involved in the shoot to root signal transduction, auxin induces proton extrusion and ferric reductase activity [35,36]. Heavy metals, particularly Fe, can catalyze the Haber-Weiss reaction, which promotes the production of active hydroxyl radicals that damage macromolecules [37,38].

To sum up all these findings, we suggest that FROs may have a diverse role, not only in iron distribution within plants and Fe uptake, but in addition they have different expression profiles under abiotic stresses, metals toxicity and hormone application. However, up to now, there has been no comprehensive study which can describe the role of rice FRO genes separately, and their regulatory mechanisms in plant growth, abiotic and metals stresses. The aim of this study is to investigate the expression pattern of the *OsFRO1* and *OsFRO7* rice gene under abiotic stresses, metals toxicity and hormone applications. Additionally, a comprehensive approach to systematic evolution, gene structure, protein organization and functional divergence was conducted to expand the understanding and evolutionary relationship of the FRO gene family and their possible mechanism. Our study will demonstrate and enlarge the knowledge about FRO genes for further functional inquiries and analysis.

## 2. Results

### 2.1. Identification, Classification, and Annotation of FRO Family Members

**Figure 1:**
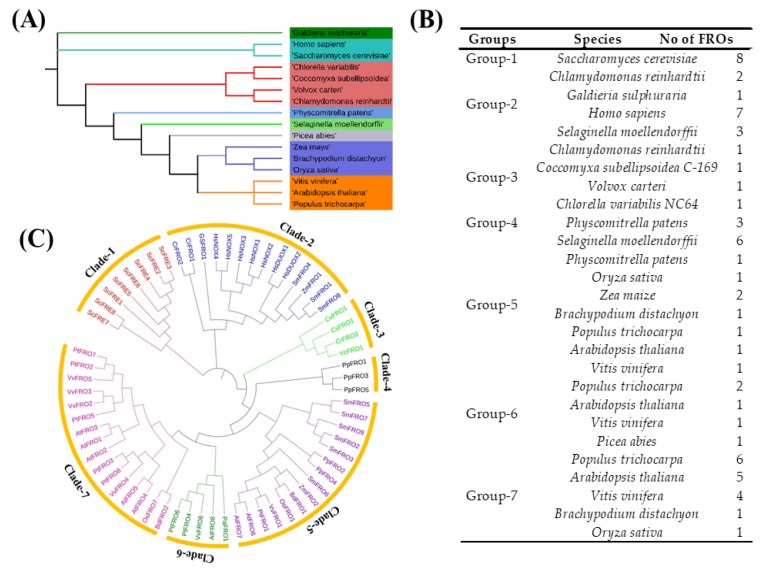
The FRO gene families. (**A**) Systematic evolutionary relationships of 16 different species among eight lineages within the subgroup. (**B**) List of FRO genes per specie within group. (**C**) The unrooted maximum-likelihood phylogenetic tree of FRO family members was inferred from the amino acid sequence alignment of FRO proteins. The seven conserved clades are marked by different colors and represented as Clade-I, Clade-II, Clade-III, Clade-IV, Clade-V, Clade-VI and Clade-VII. Scale bar represents 0.2 amino acid substitution per site.

To identify and characterize FRO genes family members in plants, the sequences were searched against the Hidden Markov Model (HMM) algorithm [39] and a total of 50 FRO genes in Plantae and 15 FRO genes in non-Plantae were retrieved. The FRO homologues genes expansively distributed among the species like in the yeast *Saccharomyces cerevisiae*, animalia, *Homo sapiens*, plants, (Eudicots), *Arabidopsis thaliana*, *Populus trichocarpa*, *Vitis vinifera*, Bryophytes, *Physcomitrella patens*, monocots, *Oryza Sativa*, *Zea mays*, *Brachypodium distachyon*, Rhodophytes, *Galdieria sulphuraria*, Chlorophytes, *Volvox carteri*, *Coccomyxa subeuipsoidea c-169*, *Chlorella variabius nc64A*, *Chlamydomonas reinhardtii*, Gymnosperms, *Picea abies*, and Lycophytes, *Selaginella moellendorffii*, (Figure 1A,B, Supplementary Information Table S1 presents database information of these particular species). The genes with alternative transcript variants of the same gene and the repeated sequence were removed. The homologous gene from different subgenomes was clustered in the same group or grouped with close similarity to gene structure, protein size or functional activity (Figure 1B). The putative functional domains of each FRO sequence was identified by using Pfam and SMART databases and structural integrity of these domains was drawn by Web Logo and EXPASY-PROSITE. All the putative FROs contain a transmembrane region with Ferric_reduc domain (Pfam accession number PF01794), and two cytosol regions, FAD_binding_8 domain (PF08022) and NAD_binding_6 domain (PF08030) (Supplementary Information Figures S1 and S2). Furthermore, the protein sequences of putative FROs (with value > 40 considered as unstable) were evaluated with EXPASY PROTOPARAM (http://www.expasy.org/tools/protparam.html) online tool for physiochemical characteristics and amino acid sequence (Supplementary Information Table S2). The deduced length of the FRO proteins and molecular weights vary widely, ranging from lowest (60.33) (*SmFRO2*) to highest (177.235) (*HsDUOX1*) amino acid residues. The FRO proteins are alkaline nature according to isoelectric point (pI), which were near to nine pI and the instability indexes of FRO proteins are mostly less than 50, which means that they all corresponded to stable proteins. Therefore, the distribution of pI of proteins has a relationship with protein length, subcellular localization, taxonomy and ecology of organisms. The majority of the proteins are all hydrophobic, as reflected by a grand average of hydropathicity (GRAVY) values. The aliphatic indexes of the FRO proteins are ranged from 82.88 to 113.37, regarding as a positive factor for the increase of thermostability of globular proteins. The major amino acid of the FRO proteins is Ser, followed by Leu, while other most abundant amino acids are Pro, Ala, Asn, or Thr, varied depending on the particular FRO protein (Supplementary Information Table S2).

### 2.2. Systematic Evolutionary Relationship, Gene Structural Diversity, and Motif Analysis

To discover the evolutionary phylogenetic relationships and functional divergence among FRO genes, FRO homologs from rice and other species were selected for the multiple sequence alignments and phylogenetic relationships analysis (Figure 1C, Supplementary Information Table S1). For an evolutionary relationship, initially, we build an unrooted maximum-likelihood phylogenetic tree using MEGA 6.06 Software. The sequences of the family-specific conserved domains of FRO proteins, namely Ferric_Reduct (PF01794), FAD_binding_8 (PF08022) and NAD_binding_6 (PF08030), were clustered into seven well-conserved clade-1-VII based on the difference of protein topological structure with high bootstrap support (Figure 1C). The tree displayed the deep duplication nodes among the FRO paralogues and the topology association within clades was highly consistent in plant species. The *FRE* genes (1–8) were clustered into clade-1 due to exon/intron structure, protein length, domain organization, and close functional homology. Correspondingly, clade-II genes have an extra NADPH_Ox domain which is responsible for abiotic stresses in plants and in the animal for pathogen attack. Whereas other plant FRO genes were clustered into clade-V and VII. Thus, concluding that all the FRO homologs within distinct clades are due to exon/intron structure, protein length, domain organization and an identical percentage of amino acids sequences.

The structural diversity of the genes and a conceivable mechanism of evolution were considered to analyze exon/intron distribution. The exon/intron organization pattern was obtained from the alignment of coding region sequences with respective genomic full-length sequences by using online GSDS (http://gsds.cbi.pku.edu.cn/) tool. The structural distribution of exon/intron of FRO genes can be split into seven well-conserved groups, which are represented as GI-GVII (Supplementary Information Figure S3). The length and number of introns in plant FRO genes are conserved but having difference among the subgroups, where intron arrangement, position, and phases is slightly less conserved in orthologous genes. Interestingly, the plant FROs have less number of exon/introns and are distributed among the subgroups (Supplementary Information Figure S3), while the non-plant type FREs (fungi) have only exons. Although ‘*NOX* like’ genes have more complicated structures because of saturated number of exons and introns, according to the phylogenetic tree (Figure 1C), all the “non” type of homologous genes were clustered in one group and plant FROs were found scattered in different groups due to the relationship of protein, size length, exon/intron distribution and functional activity.

**Figure 2:**
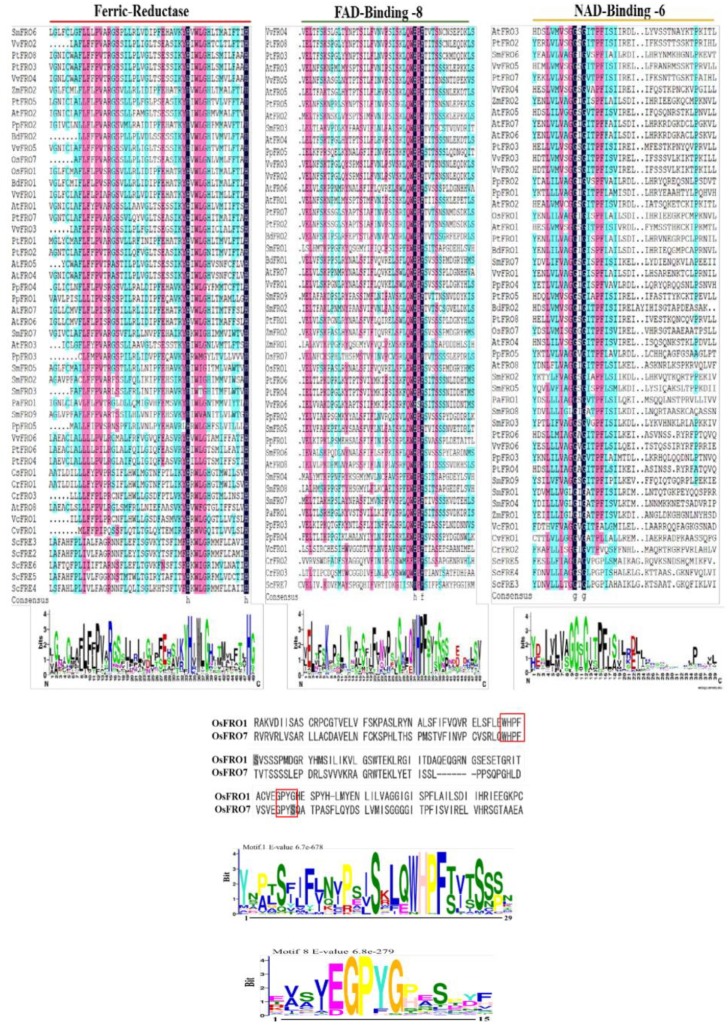
Amino acid sequences alignment analysis of Ferric_reduct, FAD_binding_8 and NAD_binding_6 domains of FRO proteins and the consensuses are shown in small letters. The two motifs HPFT in FAD binding domain and GPYG in NAD binding domain are well conserved in *OsFRO1* and *OsFRO7* protein sequences.

The conserved motifs of FRO proteins were searched through the online MEME server (Version 4.10.0, http://meme-suite.org/tools/meme) with default parameters and total twenty distinct motifs were identified and numbered from 1 to 20. The details of the putative motifs are shown in Supplementary Information Figure S4, Table S3. The conserved domains of the FRO proteins were confirmed by Pfam, SMART, Inter Pro Scan, Conserved Domain Database (CDD), NCBI (http://www.ncbi.nlm.nih.gov/cdd/), and Scan Prosite databases. The family-specific domains, Ferric_Reduct (PF01794), FAD_binding_8 (PF08022) and NAD_binding_6 (PF08030), were multiple aligned through DNAMAN software and the logos were drawn via Web Logo (http://weblogo.berkeley.edu/logo.cgi) online tool (Figure 2). The two conserved motifs, HPFT in FAD-binding domain and GPYG in NAD binding domain were quite conserved in the *OsFRO1* and *OsFRO7* genes, which are associated with co-factor binding, however, the other amino acid sequences was imperfectly conserved in both genes (Figure 2).

### 2.3. Chromosomal Location and Gene Duplication of FRO Genes

**Figure 3:**
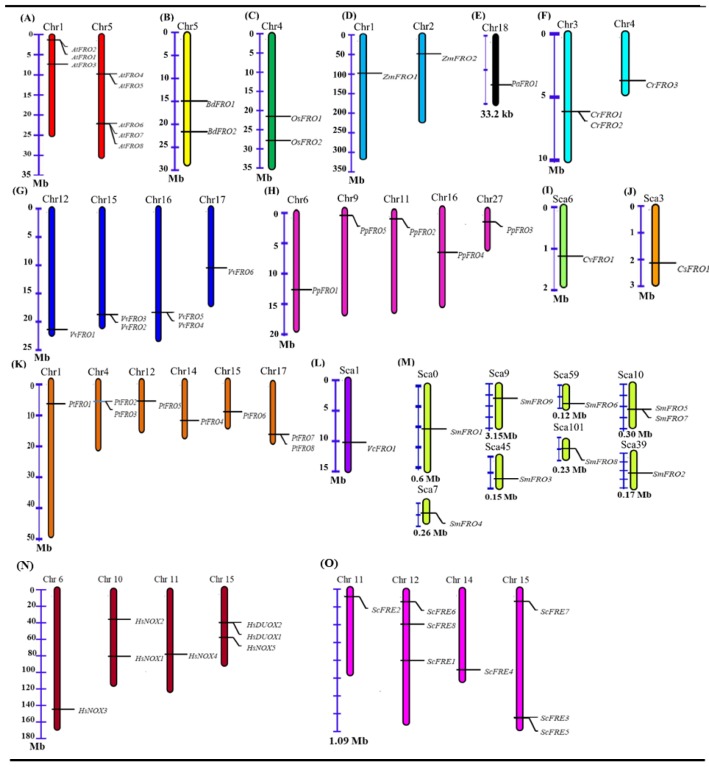
Chromosomal localization of FRO genes Families (**A**), *Arabidopsis thaliana*; (**B**), *Brachypodium distachyon*; (**C**), *Oryza sativa*; (**D**), *Zea mays*; (**E**), *Physcomitrella patens*; (**F**), *Chlamydomonas reinhardtii*; (**G**), *Vitis vinifera*; (H), *Physcomitrella patens*; (**I**), *Chlorella variabilis NC64*; (**J**), *Coccomyxa subellipsoidea C-169*; (**K**), *Populus trichocarpa*; (**L**), *Volvox carteri*; (**M**), *Selaginella moellendorffii*; (**N**), *Homo sapiens*; (**O**), *Saccharomyces cerevisiae*; respectively. The graphical view was drawn from each gene ID and scaffolds information and position of each gene are indicated by line, whereas scale bar represents the total length of chromosome.

The chromosome location and annotation information of the FRO genes in the genome showed that FRO genes are randomly distributed among the species (Figure 3, Supplementary Information Table S4), *Arabidopsis thaliana* contains eight FRO genes, among which *AtFRO1*, *AtFRO2*, and *AtFRO3* (37.5%) are located on chromosome 1 and *AtFRO4*, *AtFRO5*, *AtFRO6*, *AtFRO7* and *AtFRO8* (62.5%) are located on chromosome 5. *Brachypodium distachyon* possesses only two FRO genes (*BdFRO1* and *BdFRO2*) which are located on chromosome 5. Similarly, *Oryza sativa* also has two FRO genes (*OsFRO1* and *OsFRO7*) located on chromosome 4, and *Zea mays* genes (*ZmFRO1* and *ZmFRO2*) are located on chromosomes 1 and 2, respectively. *Vitis vinifera* holds six FRO genes of which *VvFRO1* is located on chromosome 12, *VvFRO2* and *VvFRO3* on chromosome 15, *VvFRO5* and *VvFRO4* on chromosome 16 and *VvFRO6* on chromosome 17. *Chlamydomonas reinhardtii* has three FRO genes in which *CrFRO1* and *CrFRO2* on chromosome 3 and *CrFRO3* on chromosome 4. *Physcomitrella patens* has 5 FRO genes, *PpFRO1* on chromosome 6, *PpFRO5* on chromosome 5, *PpFRO2* on chromosome 11, *PpFRO4* on chromosome 16 and *PpFRO3* on chromosome 27. *Populus trichocarpa* has eight FRO genes which are distributed as *PtFRO1* on chromosome 1, *PtFRO2* and *PtFRO3* on chromosome 4, *PtFRO5* on chromosome 12, *PtFRO4* on chromosome 14, *PtFRO6* on chromosome on 15, and *PtFRO7* and *PtFRO8* are on chromosome 17. *Selaginella moellendorffii* has nine FRO genes which are distributed as *SmFRO1* on Scaf_0, *SmFRO4* on Scaf_7, *SmFRO9* on Scaf_9, *SmFRO3* on Scaf_45, *SmFRO6* on Scaf_59, *SmFRO8* on Scaf_101, *SmFRO5* and *SmFRO7* on Scaf_10, and *SmFRO2* on Scaf_39. *Homo sapiens* has seven genes and *Saccharomyces cerevisiae* have eight genes which are FROs homologs located on four and four different chromosomes, respectively. *Volvox carteri*, *Chlorella variabilis NC64*, *Coccomyxa subellipsoidea*, and *Picea abies* contain only one FRO gene, which is located as *VcFRO1*-Scaf_1, *CvFRO1*- Scaf_6, *CsFRO1*-Scaf_3, *PaFRO1*-Ma-76199, respectively. Furthermore, FRO paralogues were searched by using Plant Genome Duplication Database (PGDD, http://chibba.agtec.uga.edu/ duplication/) [40] discovering three paralogues gene pairs of *Populus trichocarpa* and no paralogues in other species were found, suggesting that FRO family genes have undergone strong selection pressure for functional expansion (Supplementary Information Figure S5). Moreover, to explore the connotation of positive selection, approximate date of the duplication and time of divergence, the rate of nonsynonymous substitution (Ka), synonymous substitution (Ks), and the Ka/Ks ratios were calculated for the three paralogues gene pairs using the mean Ks values from T = Ks/2λ, in which the mean synonymous substitution rate (λ) for *Populus trichocarpa* is 9.1 × 10^−9^.

### 2.4. Developmental and Tissue-Specific Expression Profile of Rice OsFRO1 and OsFRO7 Genes

**Figure 4:**
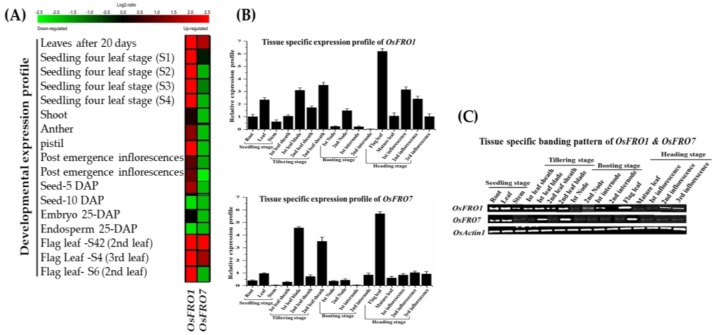
Developmental expression profile of *OsFRO1* & *OsFRO7* rice genes. (**A**) The expression profiles obtained from the database of Rice as reported by Genevestigator v3, demonstrating different expression levels of *OsFRO1* & *OsFRO7* genes in different tissues. Results were given as heat maps from green to red reflecting relative signal values; where dark green boxes represent stronger down-regulated expression and dark red boxes represents stronger up-regulation; (**B**) The graphs indicate tissue specific expression level in rice plant. The samples were collected in different developmental stages and were analyzed through qRT-PCR. Data are means represents from three independent qRT-PCR amplifications; (**C**) The tissue specific banding pattern of *OsFRO1* & *OsFRO7* rice genes through Semi-quantitative RT-qPCR analysis, *OsActineI* was used as standard control to normalized the data.

To examine the involvement of *OsFRO1* and *OsFRO7* genes in growth and development of rice, we examined the different developmental stages/tissues, a set of microarray data obtained from Genevestigator v3. The same gene expression data were also obtained from rice eFP browsers (http://bar.utoronto.ca/welcome.htm) database [41]. The expression data from the microarray analysis of rice *OsFRO1* and *OsFRO7* are presented as a heatmap from green to red (from low to high) reflecting the percentage expression (Figure 4A). The qRT-PCR and semi-quantitative RT-PCR analysis of *OsFRO1* and *OsFRO7* genes showed ubiquitous expression pattern (Figure 4B,C). The expression of *OsFRO1* gene was upregulated in leaf blade, leaf sheath, seedlings and especially in flag leaf at heading stage whereas the low mRNA accumulates were observed in other stages/tissues (Figure 4B,C). The expression of *OsFRO7* gene was highly upregulated in leaf blade, leaf sheath, and flag leaf, whereas the expression in other plant parts such as nodes, internodes and inflorescences stages were quite low. Thus, both genes have shown expression in various organs and play a vital role in plant growth and development.

### 2.5. Inducible Expression Analysis of Rice OsFRO1 and OsFRO7 Gene under Abiotic and Hormones Applications

**Figure 5:**
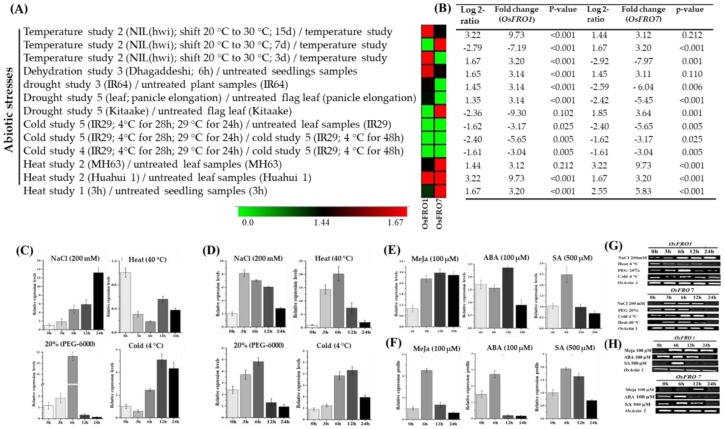
(**A**) Inducible expression profile of *OsFRO1* and *OsFRO7* in response to abiotic stresses, as reported by Genevestigator v3, demonstrating different expression levels of *OsFRO1* and *OsFRO7* genes in different tissues. Results were given as heat maps from green to red reflecting relative signal values; where dark green boxes represent stronger down-regulated expression and dark red boxes represents stronger up-regulation; (**B**) Log 2-ratio and fold changes in the expression of *OsFRO1* and *OsFRO7* genes under abiotic stresses; (**C**) Inducible expression profile of *OsFRO1* in response to treatment with NaCl, Heat, Cold and PEG (6000); (**D**) Inducible expression profile of *OsFRO7* in response to treatment with NaCl, Heat, Cold and PEG (6000); (**E**) Inducible expression profile of *OsFRO1* in response to hormones treatment ABA, MeJa and SA; (**F**) Inducible expression profile of *OsFRO7* in response to hormones treatments ABA, MeJa and SA. Mean values represents from two independent qRT-PCR amplifications; (**G**) Reverse transcriptase analysis and banding pattern of *OsFRO1* and *OsFRO7* genes in response to treatment with NaCl, Heat, Cold and PEG (6000); (**H**) RT-PCR analysis and banding pattern of *OsFRO1* and of *OsFRO7* genes under different hormones treatments. Where *OsActineI* was used as standard control to normalized the data.

To evaluate the molecular mechanism and transcriptional regulation of *OsFRO1* and *OsFRO7* against different abiotic stresses and hormonal treatment, initially, we searched the cis-elements in the promoter region of OsFROs using Plant CARE online program and responsive elements (ABRE, P-box, Sp1, GC-motif) were found, which is mainly involved in hormonal interaction and light stress (Supplementary Information Table S5). Furthermore, we analyzed the microarray data of rice via Genevestigator v3 (Figure 5A,B), which made us to analyze the expression level of these genes using qRT-PCR under environmental stresses. Therefore, we investigated the inducible expression characteristics of two candidate genes, *OsFRO1* and *OsFRO7*, rice seedlings subjected to different environmental stresses, and the different expression levels were observed at various time points (Figure 5). The FRO genes in rice exhibit response to numerous environmental stresses such as salt, heat, cold, drought and hormonal treatments like, ABA, MeJA, and SA. According to the qRT-PCR analysis, it was found that with the passage of time after NaCl_2_ (200 mM NaCl) stress, the transcript levels of *OsFRO1* were evaluated and reached up to maximum level after 24 h (Figure 5C), whereas in contrast the expression level of *OsFRO7* gene were reached to the maximum level after 3 h post treatment and then concomitant down-regulation occurred at 6 h and 12 h (Figure 5D). Similarly, the selected candidate genes were also exposed for dehydration stress by providing 20% PEG-6000 in Hoagland’s solution (Figure 5C,D). The results revealed that both *OsFRO1* and *OsFRO7* genes showed obvious changes and significant boost were noticed after 6 h treatment, further, down-regulation was observed when the plants were exposed for a long period to dehydration state (Figure 5C,D).

When chilling (4 °C) stress was applied, the results showed that both (*OsFRO1* and *OsFRO7*) genes showed a progressive increment in the expression levels and reached to the maximum level after 12 h, later exposure to cold showed a decline in expression level (Figure 5C,D). Further, the results obtained from heat stress revealed increase expression level of *OsFRO1* gene after 3 h and 6 h and then decreased were followed by 12 h and 24 h, whereas *OsFRO7* had shown no expression under heat stress (Figure 5C,D). The candidate FRO genes were also tested against hormonal stresses (ABA, MeJA and SA treatments) (Figure 5E,F). The results unveiled that in response to signaling molecules, *OsFRO1* and *OsFRO7* underwent clear changes in expression. After, the treatment with MeJA (100 μM), the simultaneous up-regulation at 6, 12 and 24 h in *OsFRO1* was noticed, whereas the *OsFRO7* gene was only up regulated at 6 h (Figure 5E,F). An increase in expression level after 6, 12 h post ABA (100 μM) treatments were noticed in the OsFRO1 and OsFRO7 genes, respectively (Figure 5E,F). Likewise, in response to SA (500 μM) significant mRNA accumulations were noticed at 6 h in both genes (*OsFRO1* and *OsFRO7*) after treatment (Figure 5E,F). Furthermore, the RT-PCR analysis also supported our results which can be seen in banding pattern when OsActin1 is used as reference gene (Figure 5G,H). Here, only rice FRO genes were examined and the results obtained here suggested that each FRO has its unique inducible expression pattern against different environmental fluctuations and thus regulating its particular functional behavior in the plant stress responses.

### 2.6. Inducible Expression Analysis of Rice OsFRO1 and OsFRO7 Genes against Different Heavy Metals Stresses

**Figure 6:**
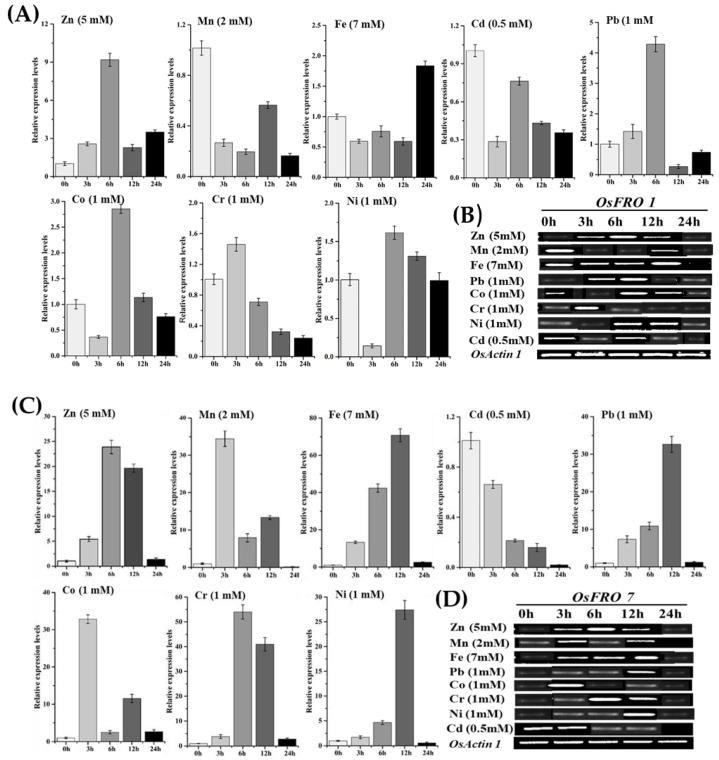
Inducible expression patterns of rice FRO family genes under different heavy metals stresses. The 18 days old rice seedlings were exposed to FeSo_4_·7H_2_O (7 mM), CdCl_2_ (0.5 mM), PbNo_3_ (1 mM), K_2_Cr_2_O_7_ (1 mM), NiCl_2_ (1 mM), MnSo_4_ (2 mM), CoCl_2_ (1 mM) and Zn(No_3_)_2_ (5 mM) treatments for 24 h. (**A**,**B**) represents the expression profile of *OsFRO1* and (**C**,**D**) represents the expression pattern of *OsFRO7.* The samples were taken at (0 h, 3 h, 6 h,12 h and 24 h) duration, RNA was extracted and analysis were performed through qRT-PCR and Semi RT-qPCR. Mean values represents from two independent qRT-PCR amplifications, where *OsActineI* was used as standard control to normalize the data.

To further insight the transcriptional regulation and expression pattern of *OsFRO1* and *OsFRO7* genes and the possible involvement of heavy metal stresses, two weeks old rice plants were exposed to eight different metals stressors such as Fe, Cd, Ni, Mn, Zn, Pb, Co and Cr (Figure 6).

The temporal induction of *OsFRO1* and *OsFRO7* genes at the transcriptional level at a various time point were assessed by real-time qRT-PCR and semi-quantitative RT-PCR analysis. Our results demonstrated that a rapid and marked increase in both genes expression occurred, which peaked after 24 h stress duration (Figure 6). Transcript induction of *OsFRO1* and *OsFRO7* genes were reached to maximum level due to Zn, Pb, Co and Ni after 6 and 12 h, respectively (Figure 6A,C), and after later expose to these stresses irregular or abrupt changes were seen in the expression of these genes (Figure 6A,C). The subjection of Cd stress downregulated the expression of both genes (Figure 6A,C). On the contrary, the expression of both genes was triggered after 12 and 24 h treatment due to induction of Fe while in early time points no such changes were seen (Figure 6A,C). Meanwhile, when we checked the expression pattern under Mn treatment, we noticed divergent results, the *OsFRO1* gene was downregulated (Figure 6A,B), while the *OsFRO7* gene was upregulated after 3 h (Figure 6C,D). Likewise, in the case of Cr, the expression of *OsFRO1* gene was immediately down-regulated after 3 h treatment (Figure 6A), whereas, the *OsFRO7* gene was upregulated by 6 and 12 h treatments (Figure 6C). Moreover, the physical appearance of young seedlings and micronutrients was also changed after heavy metal treatment. Additionally, the RT-PCR analysis also supports our results which can be seen in the banding patterns using *OsActin1* as reference gene (Figure 6B,D). Together all these outcomes indicate that both genes have complicated regulatory mechanisms under heavy metals stress and had a distinct expression pattern which provides a beneficial fundamental background and functional specificity in response to these metals stresses.

### 2.7. Determination of Antioxidant Enzyme Activities

Reactive oxygen species (ROS) are the major constituents of defense and may change the redox state of cells [42]. Plants utilize a complex antioxidant system for scavenging ROS and protect cells from oxidative damage. The antioxidant activities of plants against stress mainly depend on the plant species and metal involved. The antioxidant enzymatic activity of SOD, POD, CAT and MDA is changed under heavy metal stress in plants [43,44]. 

**Figure 7:**
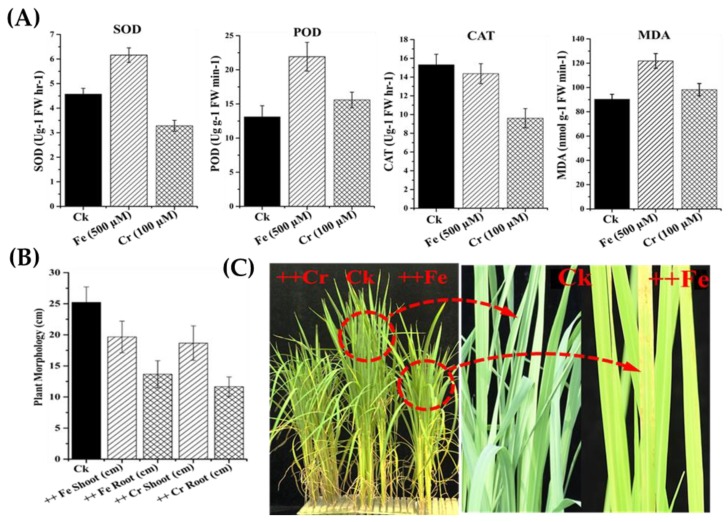
The enzymatic activity and morphological changes in rice shoots under Fe and Cr stresses. (**A**) Shows the changes in SOD, POD, CAT and MDA under Fe and Cr toxic level; (**B**) The mean of shoot length and roots length under stress conditions. The graph represents the mean of three replicates; (**C**) The physical appearance in terms of shoot (cm) and root (cm) of young seedlings and changes after heavy metal stresses after one week.

Our results indicated the significant activity of SOD, POD, CAT and MDA in leaves of rice when subjected to iron (Fe) and chromium (Cr) stress (Figure 7). The SOD, POD, CAT and MDA activity were greatly increased under Fe stress while the antioxidant activity of plants under Cr stress was decreased compared with control plants (Figure 7A). Further, the morphological characteristics (plant height and root length) of the plants were recorded under metal stress using a scale (Figure 7B), where the shoot length and root growth under Fe and Cr stress were significantly inhibited with respect to control plants (Figure 7B). The leaves of rice plants under Fe stress become brown and suffered cell necrosis, while under Cr stress the leaves were less brown although the growth was considerably retarded compared to control plants (Figure 7C).

## 3. Discussion

### 3.1. FRO Genes Expansion, Duplication, and Structural Diversity

The evolutionary relationship and gene clustering mostly fluctuate due to domain shuffling and low sequence identity between two homologous proteins. Thus, the rearrangement of domain composition, exon shuffling and gene duplication may lead to the expansion of gene families in plants during evolutionary processes [18,45]. Afterward, the duplicated genes may enhance the functional divergence, and possibly improve the functional characteristics of genes [46,47]. Moreover, single gene duplication might be a prime cause leading to the expansion of gene families in plants [25].

In this investigation, we built a phylogenetic tree based on conserved domain analysis and the proposed evolutionary relationship of the FRO gene family among different living organisms (Figure 1C). The analysis revealed that the gene architecture and domain organization of FROs are highly conserved within gene families, whereas the entire FRO genes were divided into seven conserved clades mentioned as clade-1 to clade-7 (Figure 1C). The ancient FROs proteins which were named as FREs were identified in fungi [18], these FREs perform dual functions: ROS-generation and metal ion-reduction [19]. In our study, these FRO homologs were clustered in clade-1. In a previous study on these FRE proteins they were also classified in well-conserved clades [25]. FRE protein has only exons so it may be assumed that the members of the same clade shared a similar gene structure and functional similarity in an evolutionary relationship. It has been reported that FRO proteins are the ancestral forms of *NOXs* mainly occur in animals, algae, rhodophytes and chlorophytes possessing an extra NADPH_Ox peroxidase domain functioning in the maintenance of ROS activity, both in normal and stressful conditions [25]. Therefore, the members of clade-2 display diverse exon/intron structures exhibiting even more complicated structures than plant FROs (Supplementary Information Figure S3). Furthermore, it is also suggested that plant FROs are closer in genetics to the ‘*NOX* like’ gene, so it is reasonable based on these findings that the gene duplication and fusion may be involved in the process of gene expansion. The clades-4 to 7 genes are mostly found in land plants and algae which contain the three conserved domains—Ferric_reduct, FAD_binding_8 and NAD_binding_6, but lack the NADPH_Ox domain. Therefore, separating the function of these FRO genes may affect plant physiology, growth, and development. For example, the *OsFRO1* and *AtFRO7* genes were clustered in the same clade while the function of *AtFRO7* is to direct the import of Fe to chloroplast [48]. Additionally, in rice two FRO genes which are mainly responsible for iron uptake and iron homeostasis in higher plants were located on chr.1 and 5, respectively (Figure 3) [48,49]. Therefore, it is logical that the rice FROs might have biological behavior and probably functional similarities with other clade members. The results obtained here are supported by [50], where the authors found that the same gene structure, motif and domain composition and functional similarity in MRLK family genes, which are involved in strawberry (*Fragaria vesca*) fruit ripening and abiotic stress responses, so it can be concluded that the reason for different phylogenetic clustering of FRO genes in clades-4–7 might be due to high similarities in gene structure, protein length, domain position, motif compositions and high conservation to functional activity.

The structural diversity of genes and loss or gain of an intron within multiple gene families also contribute to the evolutionary mechanism and variability [51,52]. Besides evolution, gene and domain organization also play a vital role in response to stress conditions. After examining the exon/intron structures of FRO (Supplementary Information Figure S3), we have found that the numbers of introns and intron phases are considerably conserved in plant FROs, where the position of introns are distinct among the subgroups. Therefore, the functional diversity and divergence of the FRO family proteins in plants may be due to long-term evolution history. These results are similar to those of other research studies, that were carried over the evolution mechanism of *NOX* gene families [25]. As the evolution of plants genome could be tandem, segmental or arise from the whole genome, we therefore analyzed FRO genes for gene duplication (Supplementary Information Figure S5). In our study we found three pairs of gene duplication in *Populus trichocarpa*, suggesting that the expansion of FRO genes occurred through segmental duplication and was passed on under a strong negative selection process during evolution.

### 3.2. Evolution and Functional Diversity of Conserved Domains in Plant FRO Genes

Domain architectures evolve in different modes, and thus the frequent fusion during evolution process may complicate domain composition and structural diversity in prokaryotes and eukaryotes [53]. The FRO family associates and their homologs originated from a common ancestor sharing three canonical domains, a heme-containing transmembrane region ferric reductase domain (Pfam: PF01794), and the two C-terminal cytoplasmic FAD-binding (PF08022) and NAD-binding (PF08030) domains [14,35,54], found in all living organisms from prokaryotic bacteria to eukaryotic angiosperms [25]. Ferric-reductase has the ability to transfer electrons from extracellular ferric ions to generate the reduced form of ferrous ions, which can then be transported across the plasma membrane by specific iron transporters [55,56]. The core conserved ferric-reduct domain exists in many genomes and found in multiple genes [57]. The two C-terminal domains (NADPH-binding and FAD-binding domain) expand the functional unit, in which the NADPH-binding domain provides the enzyme with a readily available electron donor, thereby raising its proficiency. Meanwhile, the FAD-binding region optimizes the energetic profile of the electron transport chain. This module with redox and electron transfer properties is beneficial to many redox systems [53]. The emergence of the ferric-reduct domain probably transfers from prokaryotes to eukaryotes in early evolution by different modules that either participate in redox systems or are regulatory components [58,59,60]. Then it is possible that the core results after independent fusion events in eukaryotes enlarge the structural complexity and function of FRO genes by adding FAD_binding_8 (PF08022) and NAD_binding_6 (PF08030) domains from rhodophytes and green algae, and then furthermore, gene duplication and gene fusion increase the number of FRO genes [53].

The specific position of conserved domains of each FRO gene was also identified in this investigation, whereas all the FROs have three highly conserved in domains construction, except the position and the distribution of amino acid residues in every domain is dissimilar in some FROs (Supplementary Information Figure S6). Three H, F and G conservative amino acid residues presented with H××××H in Ferric_redut, H××F in FAD and G×G NAD_binding domain indicated as small letters in Supplementary Section Figure S5. However, other conserved features including the FAD-binding motif (HPFT) and NAD-binding motif (GPYG) are the signature sequence in *OsFRO1* and *OsFRO7* genes and thus, are associated with co-factor binding, although the other amino acid sequence was imperfectly conserved in both genes (Figure 2).

### 3.3. Tissue-Specific Gene Expression of Rice OsFRO1 and OsFRO7 and Specific Role in Response to Metals Stress

The particular protein family members in plants have common genes expression profile characteristics. This may coordinate and/or differ in functional interaction of the family members. The previous study had shown that FRO genes in rice have clear function [16,26,61,62,63]. In *Arabidopsis*, FROs homologous genes which contribute to different biological processes and/or stress responses with obvious tissue specificity in gene expression have been functionally characterized [13,27,28]. All the FROs are expressed in different photosynthetic tissues and perform various functions like, *FRO2* reducing the Fe^3+^ to Fe^2+^ subsequently transported across the plasma membrane of root epidermal cells, *FRO4* and *FRO5* function redundantly to reduce copper to facilitate its uptake from the soil, *FRO7* is responsible for iron delivery to chloroplast and *FRO3* and *FRO8* are assumed to influence mitochondrial metal ion homeostasis [29,31]. The homologs of FROs in fungi show dual functions of both ROS-generating and metal ion-reducing [19]. While algae FROs found only functioning in ROS production and have shown no involvement in iron homeostasis [64,65]. The *LeFRO1* is expressed in roots and shoots [24], while the expression pea *FRO1* is noticed in roots (plus nodules) and leaves under iron stress [23].

The functional prediction and database searching also suggested that *OsFRO1* involvement in iron homeostasis [33] and the expression have been reported in leaves of Zn^2−^, Mn^2−^- and Cu^2−^-deficient plants [48]. The expression of *OsFRO1* gene was significantly correlated with Fe and/or Zn concentrations in seeds [66]. The regulation of rice genes related to Fe excess and deficient stress have been studied by various researchers [9,67], Hence, FRO genes are expressed in different tissues depending on the location within cell compartments and are responsible for iron homeostasis, iron uptake and iron translocation from root-to-shoot through xylem and the transport of Fe to subcellular organelles in higher plants [26,48,49,61,62,63,68]. Additionally, rice FROs subcellular localization is also yet not clear, and only the cellular function as membrane-bound protein in mitochondria was mentioned by [69], which means that rice iron uptake may differ from non-grass species [1,70]. Moreover, the low expression level of *OsFRO1* under Cr stress reduces the Cr uptake as well as uptake of iron and Fe reductase [71]. Therefore, it can be concluded that *OsFRO1* and *OsFRO7* genes play a role in heavy metal homeostasis, gene regulation, expression and plant physiology. Both genes are highly expressed in flag leaves, which are considered the key source of phloem-delivered photo- assimilated remobilized metals and have a role in the grain filling stage. We also hypothesize that *OsFRO* genes may be sensitive to some of the heavy metals, and thus it could be assumed the reduction of metals uptake and iron reductase activity, but further study is needed. The distinct expression patterns may provide a beneficial fundamental background which may open new avenues for the study of metal detoxification and individual role of each rice FRO against heavy metal stresses.

### 3.4. Inducible Expression Pattern of OsFRO1 and OsFRO7 Gene against Environmental Stresses and Hormones Treatments

Numerous adverse environmental factors such as ion toxicity, salinity, drought, extreme temperatures negatively affect plant growth and development [72,73,74,75,76,77]. Several of these abiotic stresses cause general or specific effects on growth and plant development and changes at the transcriptional level [16,78,79,80]. Here, we found that rice FROs are sensitive to a set of abiotic stresses, and their transcriptional variation was greatly influenced by salt, cold, drought, MeJA and ABA stress treatments (Figure 5C–H), indicating their participation in stress responses in rice. *Arabidopsis FRO7* gene is functionally similar to rice *FRO1* (Figure 1C), which is directs the import of Fe to chloroplasts [48]. Similarly, *AtFRO1*, a homologous of rice FROs, were also found to be induced by 1-naphthoxy-aceticacid (1-NOA) and was decreased by 1-naphthaleneacetic acid (NAA) treatment under Fe- deficient conditions [81]. Nevertheless, rice FROs are very involved in iron translocation and homeostasis but to some extent, display structural similarity except for a lack of the NADPH_Ox domain in FRO homologs ‘like *NOXs’* [18], which suggests that perhaps FROs are involved in abiotic and hormonal interactions. Some responsive elements of hormonal stresses are also found in the promoter regions of rice FRO genes (Supplementary Information Table S5). In the present study, *OsFRO1* is downregulated by high temperature treatment, whereas *OsFRO7* was upregulated (Figure 5). Maximum expression of both genes (*OsFRO1* and *OsFRO7*) was observed under salt, drought and cold stress (Figure 5C,D). Likewise, for hormone treatments (ABA, MeJa, and SA) *OsFRO1* gene was significantly upregulated, whereas *OsFRO7* was only upregulated by SA and was not affected by other treatments (Figure 5E,F). These results suggested the unique expression profile of *OsFRO1* and *OsFRO7* have different functions and mechanisms in response to stress evaluation and might play crucial roles in the plant defense system, although both genes are stress responsive and might be involved in stress signaling through a possible cross-talks linkage among the FRO gene family members.

### 3.5. Changes in Antioxidant Activity under Iron (Fe) and Chromium (Cr) Stress

The antioxidant enzymatic activity protects plant from oxidative damage by scavenging free radicals and peroxides through elevated activity under heavy metal stress [82]. In the present study the antioxidant activity of enzymes like SOD, POD, CAT and MDA significantly increased under Fe and Cr stresses. Therefore, it is suggested that leaf antioxidant activity enzymes (SOD, POD, CAT and MDA) play a significant role to cope with Fe- and Cr-induced oxidative stress in rice. Quantitative changes in antioxidant activity have also been reported in Cd^2+^ or heat, abiotic and other metal stress in barley, maize and rice plants [83,84], although, the antioxidant activity in rice under Cr stress may vary [85,86]. The morphology of the plants was also changed in response to Fe and Cr stresses as evidenced by reduced shoot length, root growth, stunted growth and low tillering. Other researchers also reported in rice that exposure to Fe and Cr stresses increase the accumulation of other metals that change the morphology of plants [87,88,89,90]. Further, the responses to these stresses compartmentalize with other stresses, so it does not overlap with one specific stress.

## 4. Materials and Methods

### 4.1. Sequences Assembly and Identification of FRO Genes Family Members

To comprehensively annotate the putative protein sequences and genomic information of FRO gene families, firstly we retrieved 50 FRO genes in 16 different species from publicly available databases. All of the retrieved protein sequences were the most recent non-redundant ones. To further authenticate the reliability of candidate proteins, we also performed a BLASTp search against the corresponding genomes by using full-length amino acid sequences, the method followed by [91]. Among those genes with alternative splice variants, the longest was chosen for further analysis. Subsequently, all the candidate protein sequences were further examined to confirm their completeness and presence of the core domain via the following online tools: SMART (http://smart.embl-heidelberg.de/) [92], Inter Pro Scan program (http://www.ebi.ac.uk/interpro/) Conserved Domain Database (CDD) (http://www.ncbi.nlm.nih.gov/cdd/), and Scan Prosite (http://prosite.expasy.org/scanprosite/). To identify the FRO genes homologs HMMER v3.0 (http://hmmer.janelia.org/) (E < 1 × 10^−5^), was used to perform using the family-specific Ferric_reduct (PF01794), FAD_binding_8 (PF08022), NAD_binding_6 (PF08030) domains. The potential chemical characteristics of FRO proteins like, isoelectric point (PI), molecular weight (kD), instability index, aliphatic index, grand average of hydropathy (GRAVY) and major amino acids of each FRO were obtained using the ExPASy proteomics server (http://web.expasy.org/protparam/) [93].

### 4.2. Phylogenetic Relationships, Exon and Intron Distribution and Conserved Motif Analysis

To further explore the evolutionary relationship of FRO gene families, the candidate FROs proteins were initially multiply aligned by using the ClustalW v2.0 online tool (http://www.ebi.ac.uk/Tools/webservices/services/msa/clustalw2_soap) and then the maximum likelyhood phylogenetic tree was constructed by using the MEGA 6.06 software package with default parameters and the reliability of interior branches was assessed with 1000 bootstrap repetitions. The exon/intron structure analysis of FRO genes were obtained through the online Gene Structure Display Server (GSDS, http://gsds.cbi.pku.edu.cn) software [94], by aligning coding sequences (CDS) to their corresponding genomic DNA sequences [95]. The protein sequences of FRO genes in each family were analyzed for motifs searching by using the MEME program (version 4.0) (http://meme.sdsc.edu/meme/cgi-bin/meme.cgi) with default parameters change to 20 conserved motifs and optimum motif width set to >6 and <200. For additional validation of FRO protein and sequence identities within alignment online tool WebLogo (http://weblogo.berkeley.edu/logo.cgi) was used to identify the maximum occurrence of amino acids. The logos of conserved domains annotation were performed using SMART (http://smart.embl-heidelberg.de/) and Pfam (http://pfam.xfam.org/search). The three conserved domain motifs, namely Ferric_reduct, FAD_binding_8 and NAD_binding_6 in each FRO sequence, and domain diagrams were generated by PROSITE (http://prosite.expasy.org/mydomains/).

### 4.3. Determining Chromosomal Location and Gene Duplication

The distribution of FRO genes on chromosomes was explored by using chromosomal location and annotation information from scaffolds and Gene translation ID form publicly available databases and Map Draw software [96] was used to display precise gene locations according to the physical positions. Gene duplication analysis was also conducted in FRO gene families by using the Plant Genome Duplication Database (PGDD, http://chibba.agtec.uga.edu/duplication/index/locus). Nonsynonymous (Ka) and synonymous (Ks) rates (Ka/Ks) were also calculated based on the results.

### 4.4. Cis-Regulatory Elements in the Promoter Region of FRO Genes

The 2 kb upstream region from the transcription start site of all FRO genes were obtained from their corresponding databases and were searched for the *cis*-regulatory elements by using the Plant CARE program (http://bioinformatics.psb.ugent.be/webtools/plantcare/html) [97]. Further validation of the results was cross-checked by using the software PLACE (http://www.dna.affrc.go.jp/PLACE/) [98] and Neural Network Promoter Prediction (http://promotor.biosino.org/) (Supplementary Table S5).

### 4.5. Plant Material and Growth Conditions

Rice (*Oryza sativa* L cv. *Nipponbare*) seeds were obtained from the State Key Laboratory of Crops Stress Biology for Arid Areas (Northwest A&F University, Yangling, China). The seeds were disinfected with 0.5% (*w*/*v*) sodium hypochlorite (NaClO) for 4 h, rinsed thrice with distilled water and soaked in water for 48 h in darkness. Afterwards, the seeds were germinated on moist cheesecloth at 28 °C for 72 h and wetted with deionized water each day. Only healthy and uniform seedlings were grown in hydroponic solution prepared in Milli-Q water [99], containing 16 mM, KNO_3_, 6 mM, Ca (NO_3_)_2_·4H_2_O, 4 mM NH_4_H_2_PO_4_, 2 mM, MgSO_4_·7H_2_O, 50 µM, KCl, 25 µM, H_3_BO_3_, 25 µM, Fe-EDTA, 2 µM, MnSO_4_·4H_2_O, 2 µM, ZnSO_4_, 0.5 µM, Na_2_MoO_4_·2H_2_O and 0.5 µM, CuSO_4_·5H_2_O. The plants were floated in nutrient solution fixed with foam plugged in vessels (one plant in single vessel). The nutrient solutions were continuously aerated and the environment were strictly controlled in growth chamber condition at (16 h/8 h day/night, temperature cycle of 30 °C/25 °C, 800 μmol m^–2^ s^–1^ light intensity and 60–65% relative humidity level). The hydroponic solution was changed after 24 h duration and the pH were adjusted to 5.8 by using NaOH or HCl.

### 4.6. Stress Treatments and Sample Collection

To investigate the inducible expression profile of rice FRO genes the young fully expanded seedlings (two weeks old) were subjected to various abiotic stresses, phytohormones and toxic metal exposure. For dehydration 20% polyethylene glycol (PEG-6000), solution was purified by passing it through an ion exchange column to remove any impurities and was filtered using Miracloth (22–25 μm, (Thomas Scientific, Swedesboro, NJ, USA). Salt (200 mM NaCl) were prepared from stock solution by dissolving in water. For cold (4 °C) was carried out in cold cabinet, and heat (40 °C) with a white cool florescent light chamber. For hormones treatments: abscisic acid (ABA) (100 µM), methyl jasmonate (MeJA) (100 µM) and salicylic acid (SA) (500 µM) were dissolved in ethanol and stock solutions were prepared further, adjusted to final concentrations by adding an aqueous solution containing the wetting agent Tween 20 at 0.05% (*v*/*v*) and sprayed on two weeks old rice leaves. For metals treatments, FeSO_4_ (7 mM), CdCl_2_ (0.5 mM), PbNO_3_ (1 mM), K_2_Cr_2_O_7_ (1 mM), NiCl_2_ (1 mM), MnSO_4_ (2 mM) and CoCl_2_ (1 mM) were prepared from stock solutions and applied into fresh nutrient solution as mention in Section 4.5 and as [100] with exception of phosphorus (P) that prevent precipitation of Pb, following [101]. All the chemicals used for treatments were purchased from Sigma-Aldrich (Burlington, MA, USA). The whole leaf blades from all treatments were collected with four (0, 3, 6, 12 and 24 h) time points from two weeks old rice plants. For the analysis of the tissue-specific expression profiles of rice FRO genes, rice plants were allowed to grow in normal condition and different plant organs at developmental stages including seedling, tillering, booting and heading stages, were collected. The samples were immediately frozen in liquid nitrogen after harvested and stored at −80 °C until for further analysis. Total RNAs was extracted from 0.5 g from whole leaf blade sample by using RNAiso TM Plus (Takara, Dalian, China) and quality of RNA was checked by nanodrop. The final value was calculated and adjusted up to 2000 ng/µL. The cDNA was synthesized by Super Mix (Trans Gen Biotech, Beijing, China) with DNA remover according to manufactured protocol and the final volume of cDNA was obtained up to 300 ng/µL, which was further diluted with TE (Tris EDTA) buffer up to 50 ng/µL and used for qRT-PCR analysis. For RT-qPCR analysis cDNA was diluted upto 100 ng/µL. The gene-specific primers were designed by avoiding the conserved region by using Premier 6.0 software (http://www.premierbiosoft.com/primerdesign/index.html) and the rice *OsActin1* (GeneBank accession number: KC140126) was used as the internal control for analysis. The results were presented in graphs, banding pattern and/or also table lists.

### 4.7. Antioxidant Enzyme Activities under Metal Stresses

The enzymes CAT, POD, SOD and MDA were extracted from shoots of 2-week-old plants. The details of the methods of seed germination and seedling maintenance in nutrient solution were described in Section 4.5. Fe and Cr stresses were induced through addition of Fe (500 µM) and Cr (100 µM), respectively, to treatment solutions for one week and antioxidant activity was determined. More briefly, 0.5 g leaf tissue was homogenized in 5 mL phosphate buffer (100 mM), after centrifugation for 10 min the supernatant was transferred in Eppendorf tubes. For CAT analysis, the reaction mixture (2 mL) contained 100 mM potassium phosphate buffer (pH 7.0), 400 µL 6% H_2_O_2_ and 100 µL shoot extract and the decrease in absorbance was quantified at 240 nm (extinction coefficient of (36 M^−1^ cm^−1^) using a UV spectrophotometer at 30 s intervals up to 1 min [102]. The peroxidase POD activity was quantified using the method [103]. The reaction mixture (2 mL) contained 100 mM potassium phosphate buffer (pH 6.5), 1 mL 0.05 M pyrogallol solution, 400 µL 200 mM and 100 µL shoot extract. The changes in absorbance were recorded at 430 nm (extinction coefficient 12 mM^−1^ cm^−1^) in a spectrophotometer from 30 s up to 1.5 min and POD activity was calculated. For SOD activity was performed according to the method of [104] using nitro blue tetrazolium (NBT), the mixture contained comprised of 1.5 mL of 0.1 M phosphate buffer (PB, pH = 7.5), 0.3 mL of 1.3 M methionine, 0.3 mL of 750 µM NBT, 0.3 mL of 100 µM EDTA-Na_2_, 0.3 mL of riboflavin and 0.25 mL of distilled water. A total volume of 3.0 mL of assay mixture was reached by adding 0.05 m of enzyme extract and color changes in absorbance were recorded by spectrophotometer at A_560_ nm. For MDA analysis, the method of [105] was used. Leaf tissue (0.5 g) were homogenized in 5 mL of 5% trichloroacetic acid, then centrifuged at 4000× *g* for 10 min at 25 °C and 3 mL of 2-thiobarbituric acid in 20% trichloroacetic acid was added to a 2 mL aliquot of the supernatant. The absorbance was recorded at 532 nm, further correction was mad by subtracting non-specific turbidity at 600 nm absorbance and MDA concentration was recorded by extinction coefficient MDA (ε = 155 μM^−1^ cm^−1^).

### 4.8. qRT-PCR Analysis

The real-time qPCR was performed with Platinum SYBR Green qPCR Super Mix-UDG with ROX (Invitrogen, Carlsbad, CA, USA) on CFX96™ Real-Time PCR Detection System (BioRad, Foster City, CA, USA). Each reaction was consisted of 5 μL SYBR premix ExTaq (Takara, Kyoto, Japan), 2 μL cDNA samples, and 0.5 μL of each primer (10 μM) and 2 μL ddH_2_O in a reaction system of 10 μL. The thermal cycle was as follows: 95 °C for 3 min, followed by 40 cycles at 94 °C for 15 s, 62 °C for 20 s, and 72 °C for 20 s. Melting-curve analysis was performed directly after real-time PCR to verify the presence of gene-specific PCR products. This analysis was done by 94 °C for 15 s, followed by a constant increase from 60 to 95 °C at a 2% tamp rate. qRT-PCR analysis was repeated twice for further validation of results. The *OsActin1* gene was used as internal control and relative expressions were compared with that of control plants and served as a standard gene for normalizing all mRNA expression levels. The detailed list of primers was shown in Supplementary Table S6. The relative amount of template present in each PCR amplification mixture was evaluated by using the 2^−ΔΔCt^ method. The RT-qPCR analysis was performed and run on gel electrophoresis with standard markers. All the primers were designed from FRO genes sequences using Primer 6.0 (Supplementary Table S6). Each primer pair was tested via standard RT-PCR to check the size specificity of the amplified product by 1% agarose gel electrophoresis. The images were taken by gel doc system and the banding patterns were recorded. The gel photos were adjusted using the image J software (NIH, Bethesda, MD, USA).

### 4.9. Statistical Analysis

Statistical analysis was performed by using variance (ANOVA) and the means were compared by the T-test at the 5% level using the SPSS 11.5 software package (SPSS Inc., Chicago, IL, USA). Experiments performed in this study had at least three independent replications for each sample. Further, graphical presentation was prepared using GraphPad Prism 6 (GraphPad Software, Inc., La Jolla, CA, USA).

## 5. Conclusions

The results of our experiments suggesting that rice FRO genes play a significant role in many metabolic processes and the intricate signaling networks in response to a number of abiotic stresses, metals toxicities and hormonal applications. However, the particular molecular mechanism and function of rice FROs are still ambiguous. Moreover, the interactions of FROs are also poorly described. The diverse expression pattern of rice FRO genes has been noticed within tissues and changes in environmental conditions, such as drought, heat, salt, metals, and phytohormones, signifying the varied functions and unique expression pattern of these genes in the plant development and stress responses. Thus, the dissimilar changes in expression level in the same rice FRO gene against various environmental fluctuations infer a difference in their regulatory mechanism. The results obtained here shed light and clarify the background for further experiments, defining the specific role of each FRO gene in regulatory stress responses as well as in transgenic rice to unveil the function and the possible cross-talk between rice FRO proteins in plants.

## Supplementary Materials

The following are available online. Figure S1: 3D general representation of functional domains, Table S1: Detail of plant genomes analyzed in this study, Table S2: The detail information about physiochemical characteristics of FRO gene families, Table S3: Motif sequences identified by MEME tools, Table S4: Genomic information of FRO genes, Table S5: The cis-elements regulatory elements in the promoter region of *OsFRO1* & *OsFRO7* genes, Table S6: The detailed list of primers used for expression analysis.
