# Supplementary material for: Comparative in Silico Analysis of Ferric Reduction Oxidase (FRO) Genes Expression Patterns in Response to Abiotic Stresses, Metal and Hormone Applications
Source: Molecules. 2018 May 12;23(5):1163. doi: 10.3390/molecules23051163 (PMC6099960; doi:10.3390/molecules23051163)
Supplement: Supplementary file 1 [file molecules-23-01163-s001.pdf]

Supplementary Information

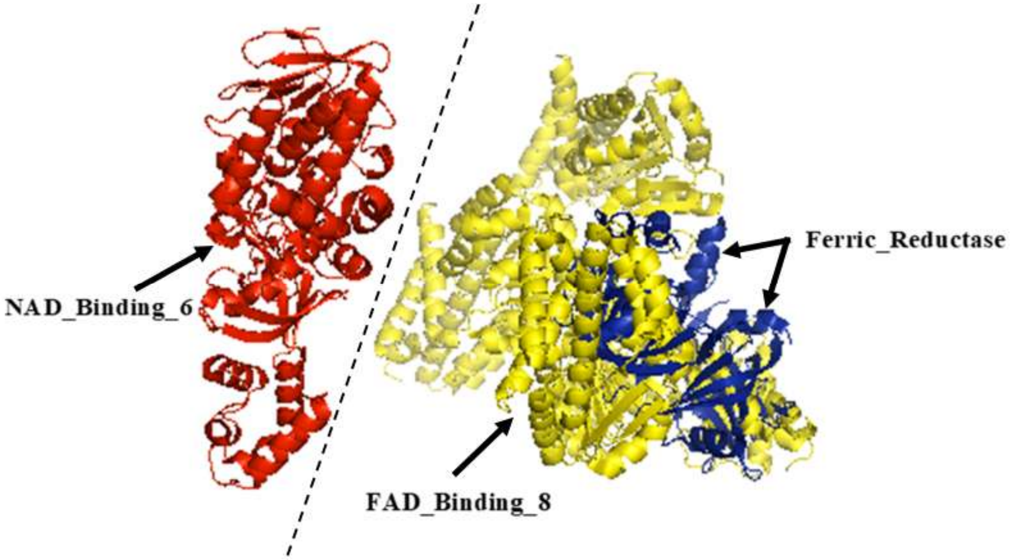

Figure S1. 3D general representation of functional domains, Red represents NAD_binding_6 domain, the Yellow represents the FAD_binding_8 domain and the Blue represents the Ferric_Reductase domain. Moreover, the Ferric_reductase and FAD_bing_8 domains (Blue+Yellow) are more highly conserved domains which are closer to each other in FRO gene families.

Name	Ferric	FAD-8	NAD-6
SmFRO1	217-365	407-529	442-560
SmFRO2	15-132	166-270	535-689
SmFRO3	164-276	310-412	422-507
SmFRO4	120-266	309-427	421-537
SmFRO5	166-283	322-412	433-607
SmFRO6	147-264	304-395	421-504
SmFRO7	150-265	301-396	404-483
SmFRO8	47-195	246-319	334-525
SmFRO9	147-263	299-393	402-507
BdFRO1	186-304	338-446	455-536
BdFRO2	189-299	330-433	442-560
ZmFRO1	148-297	340-441	447-606
ZmFRO2	198-316	350-454	463-542
AtFRO1	176-294	325-428	434-488
AtFRO2	186-303	334-449	441-560
AtFRO3	203-317	348-449	455-568
AtFRO4	159-275	311-406	412-498
AtFRO5	120-233	269-364	370-456
AtFRO6	181-299	330-440	449-530
AtFRO7	190-308	342-449	458-539
AtFRO8	162-280	316-416	422-498
ScFRE1	261-374	411-519	528-665
ScFRE2	283-396	430-532	537-691
ScFRE3	283-396	430-532	537-691
ScFRE4	276-389	424-525	530-699
ScFRE5	277-388	424-526	531-674
ScFRE6	290-402	438-544	550-692
ScFRE7	164-278	328-414	423-596
ScFRE8	104-219	253-365	372-557

Protein Structure

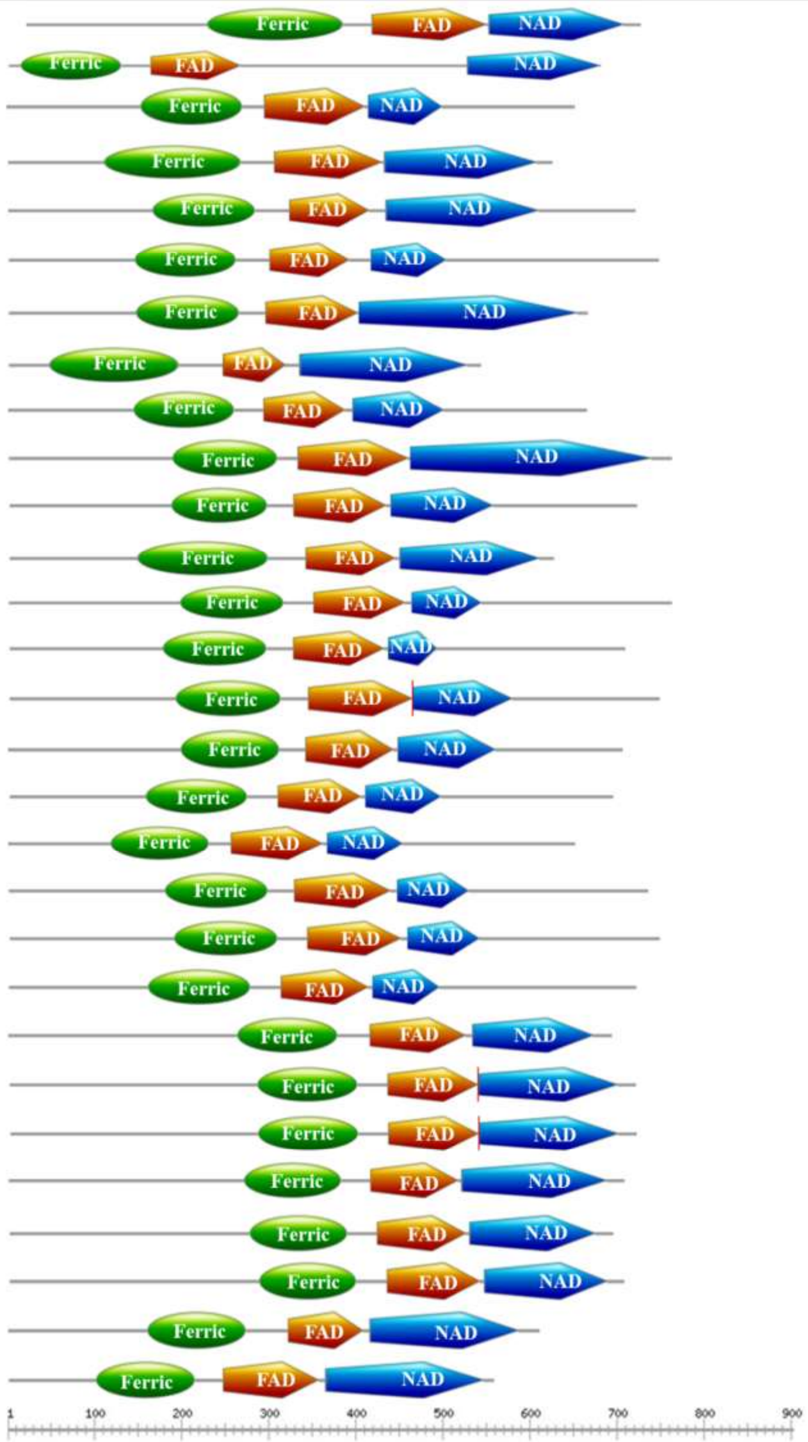

Name	Ferric	FAD-8	NAD-6
CrFRO2	122-235	277-367	375-421
CrFRO3	311-424	496-625	644-746
CsFRO1	211-328	401-454	704-749
VcFRO1	345-458	525-605	616-701
PpFRO1	159-276	307-412	421-502
PpFRO2	190-308	348-441	450-529
PpFRO3	225-334	369-472	481-564
PpFRO4	185-301	334-435	444-524
PpFRO5	179-293	327-431	439-523
PaFRO1	238-312	378-480	486-569
OsFRO1	194-312	346-450	459-539
OsFRO2	36-147	180-281	288-369
PtFRO1	180-298	332-437	446-703
PtFRO2	172-288	323-442	450-529
PtFRO3	162-278	317-409	481-564
PtFRO4	162-280	316-419	444-524
PtFRO5	167-284	317-416	439-523
PtFRO6	162-280	316-419	421-502
PtFRO7	173-289	324-422	450-529
PtFRO8	151-267	306-398	404-504
VvFRO1	184-302	336-440	449-530
VvFRO2	184-297	331-429	435-555
VvFRO3	163-276	310-408	414-527
VvFRO4	641-760	791-890	896-1012
VvFRO5	190-307	340-439	445-525
VvFRO6	157-275	311-412	418-531
CvFRO1	195-307	379-417	452-499
CrFRO1	211-328	401-454	704-749

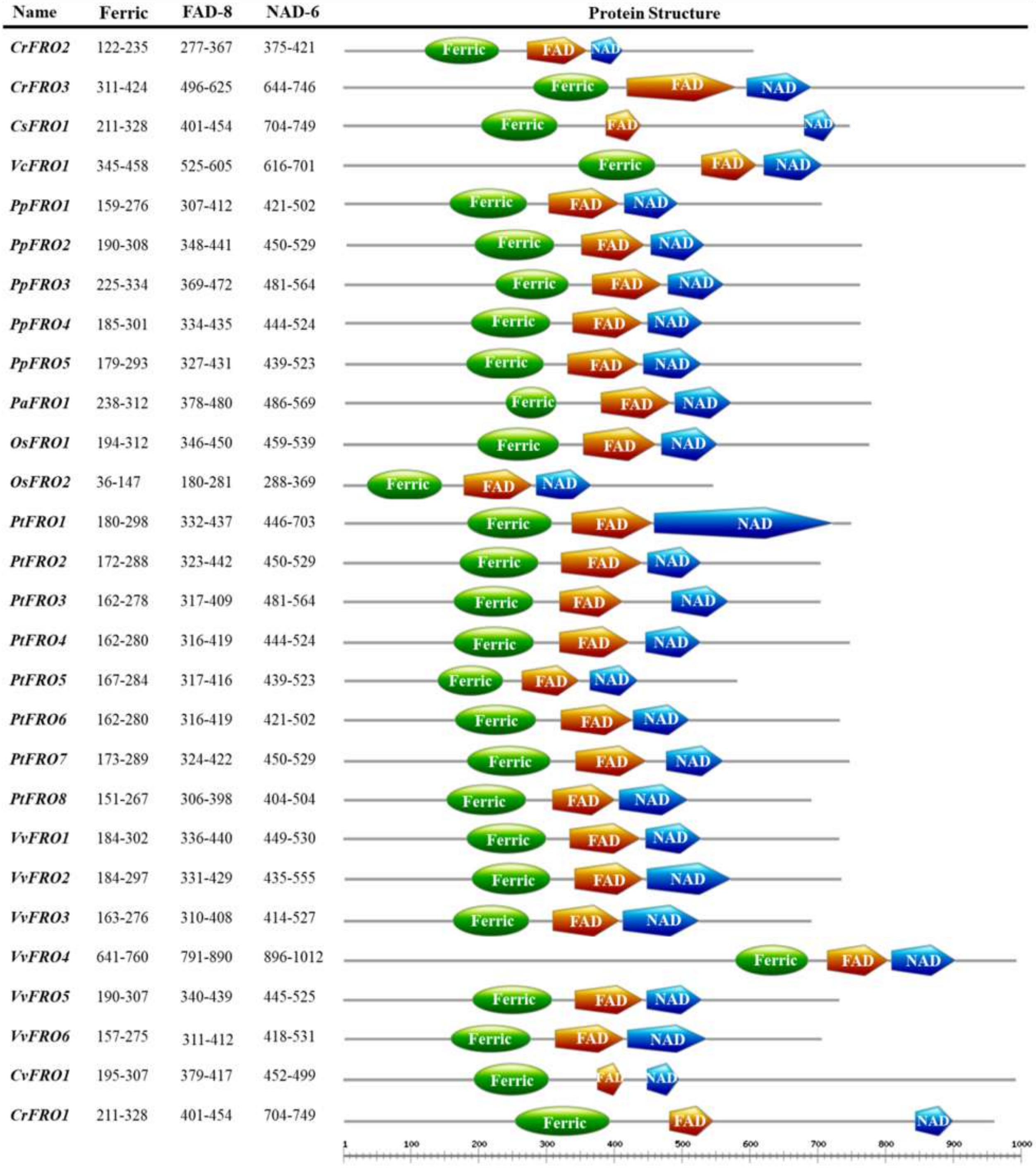

Figure S2. The Structure of the FRO proteins, Numbers indicate amino acid position of the corresponding conserved domains. The green, orange and blue ovals and pentagons indicate the Ferric means (Ferric-reductase), FAD means (FAD-binding-8), and NAD means (NAD-binding-6 domain), respectively, the scale bar represents the number of amino acids coded by each gene.

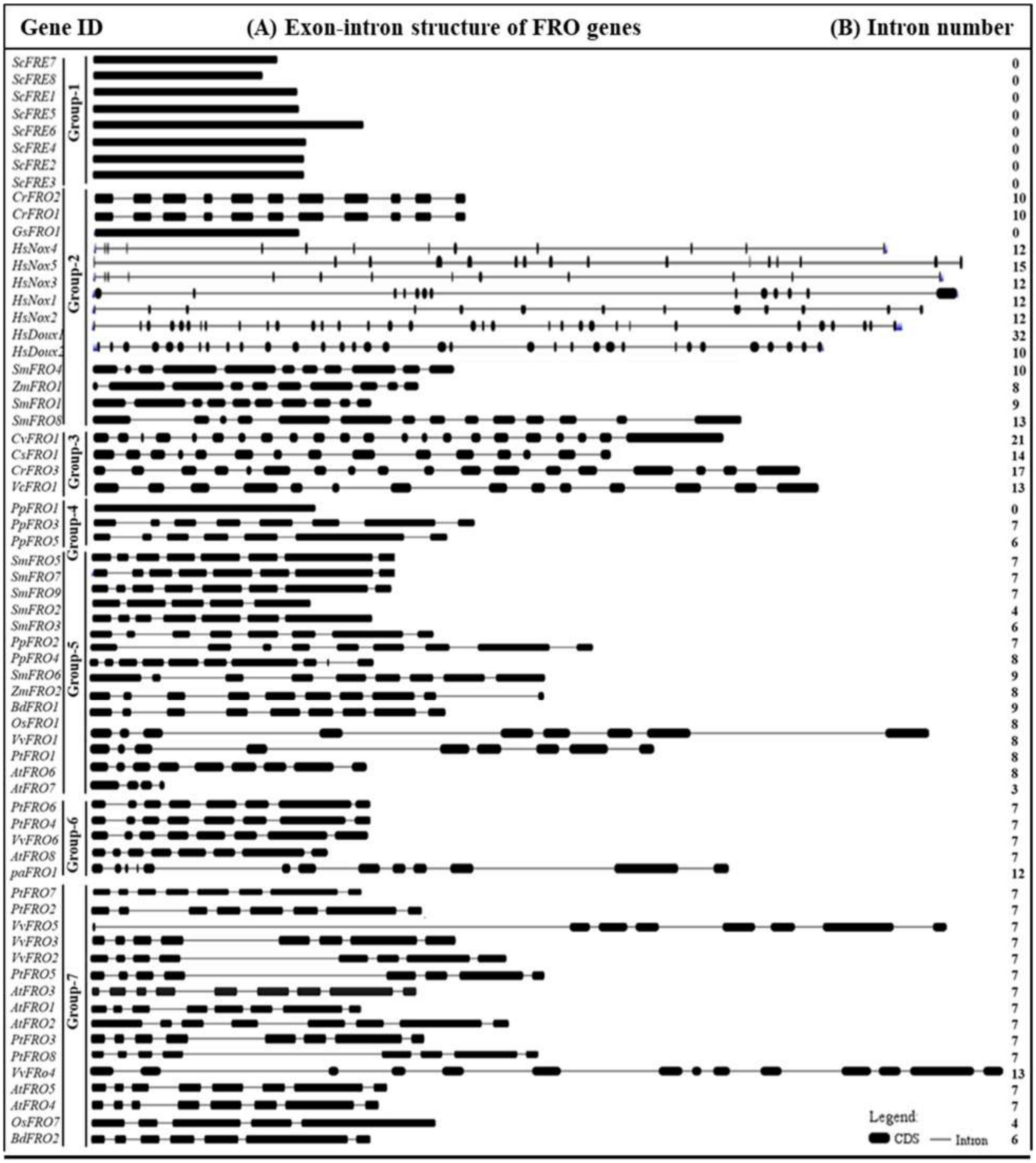

Figure S3. The exon/intron structures of FRO family genes. The structure of individual *FRO* gene was obtained through the Gene Structure Display Server (http://gsds. cbi.pku.edu.cn) by aligning the coding or cDNA sequences with their corresponding genomic DNA sequence. Black boxes represent exons and black lines represent introns. (B) represents intron numbers of each gene contained.

Site of amino acids and motif location of FROs protein

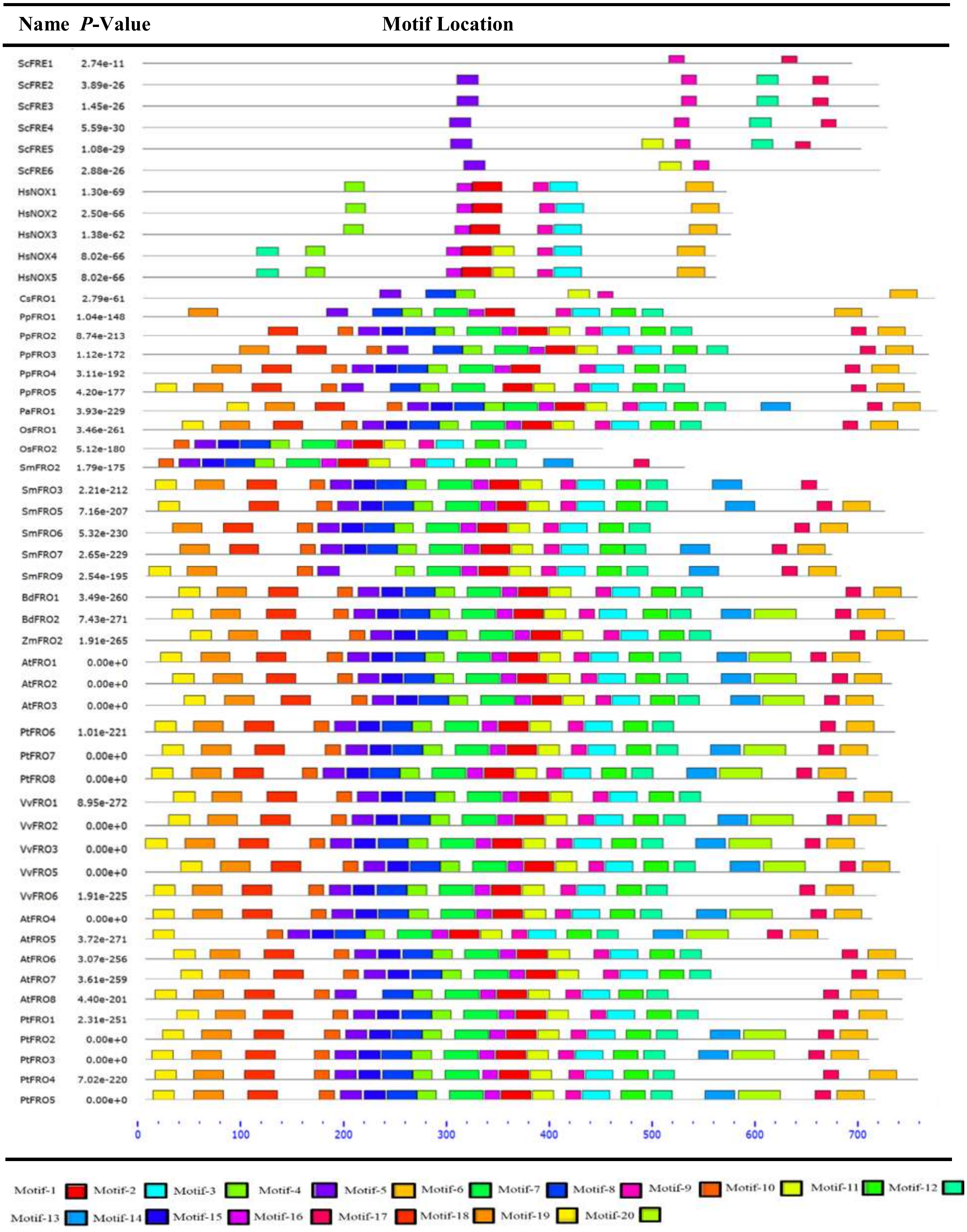

Schematic diagram of amino acid motifs of FRO, s proteins

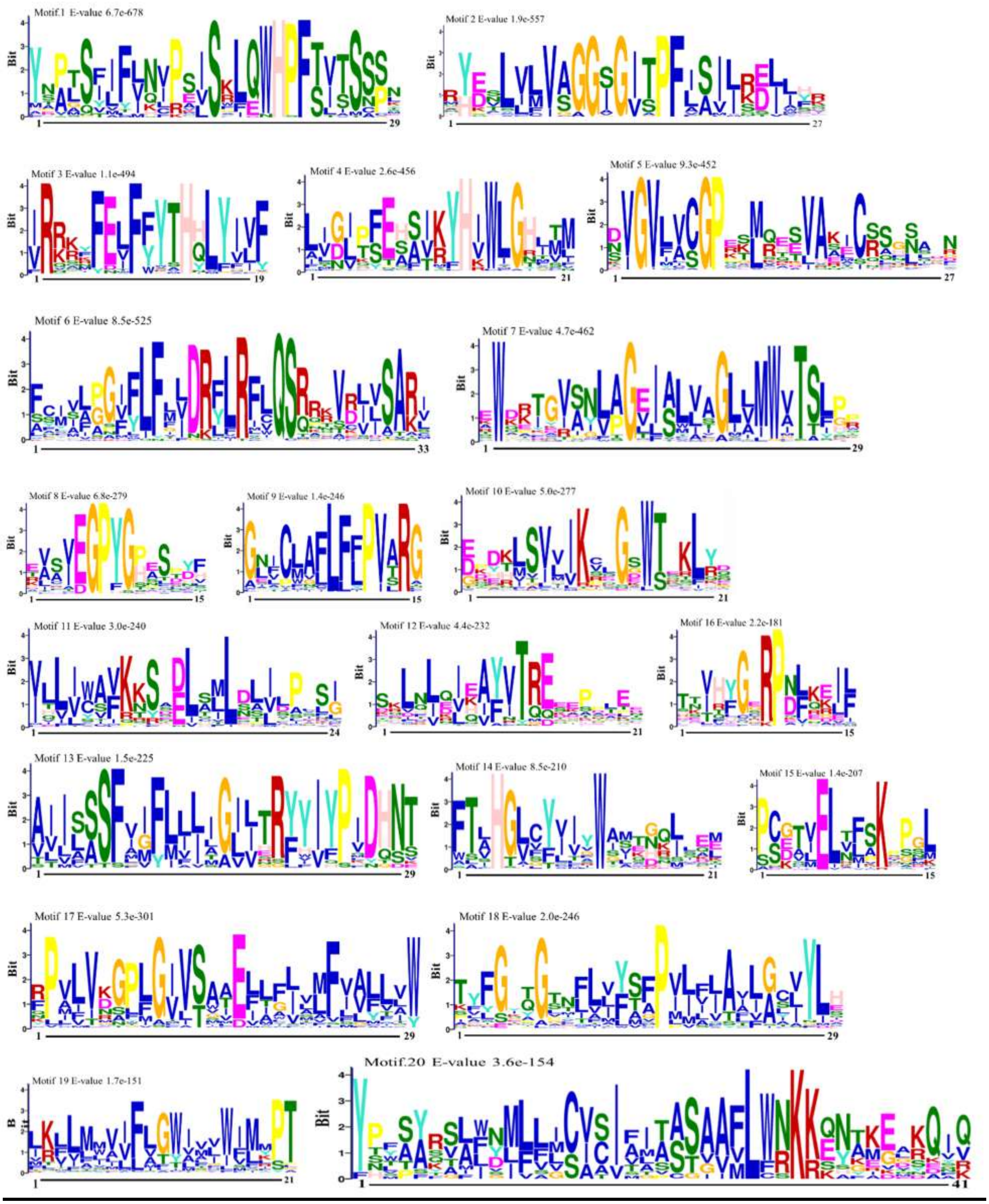

Figure S4. Phylogenetic relationships and schematic diagram of amino acid motifs of FRO, s proteins. The number of nodes represent the bootstrap value.

A

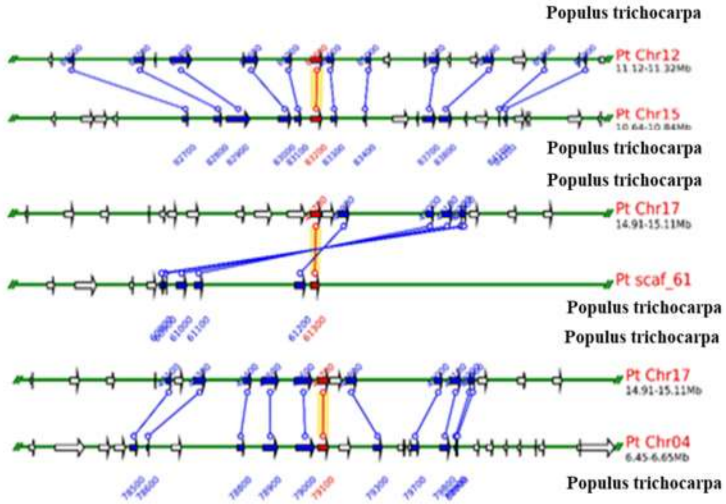

B

Ka	Ks	Ka/Ks	Duplication time billion years Ago (Mya)
0.07	0.27	0.26	1.2285
0.07	0.25	0.2	1.1375
0.41	1.25	0.32	5.6875

Figure S5. The non-synonymous (*Ka*) and synonymous (*Ks*) were estimated for the paralogous gene pairs of FRO family genes. Genome Duplication Database, Divergence time (T, million years ago, Mya) for each paralogous gene pair was calculated using the average Ks of λ substitutions of Populus trichocarpa per synonymous sites per year as T =Ks/2λ ($\lambda = 9.1\times10^{-9}$)

Supplementary Tables

Table S1. Detail of Plant Genomes Analyzed in This Study

Species	Genome size (Mbp)	Database
Homo sapiens	3400	http://asia.ensembl.org/Homo_sapiens/Info/Index
Arabidopsis thaliana	157	http://www.arabidopsis.org/
Oryza sativa	430	http://rice.plantbiology.msu.edu/index.shtml
Zea maize	2,400	http://www.maizesequence.org/index.html
Brachypodium distachyon	355	http://www.phytozome.net/brachy.php
Selaginella moellendorffii	110	http://genome.jgipsf.org/Selmo1/Selmo1.home.html
Populus trichocarpa	500	http://genome.jgi-psf.org/Poptr1_1/Poptr2_2.home.html)
Vitis vinifera	500	http://www.genoscope.cns.fr/externe/GenomeBrowser/Vitis/
Physcomitrella patens	500	http://genome.jgi-psf.org/Phypa1_1/Phypa1_1.home.html
Volvox carteri	140	http://genome.jgi-psf.org/Volca1/Volca1.info.html
Coccomyxa subellipsoidea C-169	48.82	http://genome.jgipsf.org/Coc_C169_1/Coc_C169_1.home.html)
Chlamydomonas reinhardtii	120	http://genome.jgi-psf.org/Chlre4/Chlre4.info.html
Chlorella variabilis NC64	46.15	http://genome.jgi-psf.org/ChlNC64A_1/ChlNC64A_1.home.html)
Picea abies	20,480	http://congenie.org/start
Saccharomyces cerevisiae	12	http://www.yeastgenome.org/download-data
Galdieria sulphuraria	13.7	http://genomics.msu.edu/galdieria/

The detail information about physiochemical characteristics of FRO Gene families

Gene name	Gene locus ID	CDS length	Pl	M.W (KDa)	pIs	Ii	Ai	Major amino acids	GRAY
SmFRO1	e_gw1.0.1110.1	2136	711	80.96	8.96	44.51	82.88	L (9.6%), A (8.3%), S (7.6)	-0.122
SmFRO2	e_gw1.39.66.1	1590	529	60.33	9.54	36.32	102.63	I (9.8%), L (9.1%), V (7.8%)	0.209
SmFRO3	e_gw1.45.93.1	1992	663	74.93	9.59	35.68	105.44	L (12.2%), A (8.1%), V (8.0%)	0.337
SmFRO4	estExt_fgenesh1_pm	1878	625	71.59	9.27	38.37	90.59	L (9.3%), V (8.3%), G (6.6%)	-0.024
SmFRO5	estExt_fgenesh2_pg	2157	718	80.32	9.30	40.55	105.58	L (11.1%), A (9.9%), I (8.2%)	0.291
SmFRO6	estExt_fgenesh2_pg	2271	756	84.38	8.74	39.68	108.28	L (13.2%), V (8.7%), S (7.7%)	0.399
SmFRO7	estExt_Genewise1	2004	667	74.65	9.43	39.45	107.06	L (11.7%), V (10.2%), S (8.1%)	0.349
SmFRO8	fgenesh1_pm	1628	542	61.89	9.43	39.25	94.78	L (10.7%), V (8.1%), A (7.2%)	0.160
SmFRO9	gw1.9.156.1	2028	676	76.91	9.26	42.02	103.98	L (10.2%), V (8.3%), I (8.3%)	0.270
BdFRO1	Bradi5g11147.1	2253	758	81.68	7.01	38.74	104.17	L (12%), A (10.7%), G (8.8%)	0.410
BdFRO2	Bradi5g19150.1	2187	735	80.15	9.15	40.28	102.45	L (12.9%), A (8.8%), S (8.8%)	0.330
ZmFRO1	GRMZM2G089291-T01	2517	624	71.15	9.36	41.81	93.24	L (9.3%), V (8.2%), I (6.9%)	0.090
ZmFRO2	GRMZM2G068557-T01	2926	760	84.04	7.93	41.44	103.91	L (12.4%), A (9.3%), V (9.6%)	0.398
AtFRO1	AT1G01590.1	2642	704	79.60	9.56	41.78	105.04	L (10.9%), S (10.8%), V (7.7%)	0.311
AtFRO2	AT1G01580.1	2635	725	81.50	9.37	39.61	101.81	L (11.3%), S (10.6%), I (9.1%)	0.253
AtFRO3	AT1G23020.1	2154	717	80.93	9.79	38.06	106.03	L (12.0%), S (11.2%), I (8.8%)	0.328
AtFRO4	AT5G23980.1	2337	699	80.25	9.44	39.86	101.86	L (12.0%), S (9.9%), V (7.6%)	0.183
AtFRO5	AT5G23990.1	1974	657	74.89	8.94	42.62	94.73	L (10,2%), S (10.2%), V (7.6%)	0.098
AtFRO6	AT5G49730.1	2675	738	83.45	7.94	37.76	113.37	L (13.1%), S (8.8%), V (8.7%)	0.431
AtFRO7	AT5G49740.1	2640	747	84.12	6.82	35.17	112.52	L (13.0%), V (9.4%), S (9,0%)	0.432
AtFRO8	AT5G50160.1	2520	728	83.23	9.58	47.28	110.05	L (13.0%), I (8.5%), S (7.8%)	0.281
PtFRO1	Potri.001G079000.1	2190	729	81.80	6.68	39.45	112.78	L (13.3%), S (8.9%), V (8.4%)	0.417
PtFRO2	Potri.004G079100.1	2118	705	79.45	9.34	43.35	101.53	L (11.5%), S (11.2%), I (8.4%)	0.243
PtFRO3	Potri.004G079200.1	2091	696	79.30	9.30	41.66	104.48	L (12.5%), S (9.2%), V (7.3%)	0.314
PtFRO4	Potri.012G084800.1	2232	743	83.14	9.48	47.71	112.45	L (12.8%), S (10.0%), V (7.3%)	0.394

PtFRO5	Potri.014G088000.1	2109	702	78.44	9.64	34.56	107.22	L (13.0%), S (9.8%), I (8.3%)	0.334
PtFRO6	Potri.015G083200.1	2169	722	81.10	9.43	47.48	107.76	L (12.7%), S (10.4%), I (8.2%)	0.296
PtFRO7	Potri.017G142700.1	2121	706	79.09	9.10	45.01	104.55	L (12.2%), S (10.9%), I (7.8%)	0.328
PtFRO8	Potri.017G142800.1	2058	685	78.05	9.29	41.95	105.58	L (12.8%), S (8.9%), V (7.4%)	0.311
VvFRO1	GSVIVT01023105001	2483	735	83.33	6.24	44.62	113.57	L (13.5%), S (8.4%), V (8.0%)	0.455
VvFRO2	GSVIVT01026991001	2325	714	79.56	9.68	40.87	106.90	L (13.0%), S (10.5%), I (7.6%)	0.277
VvFRO3	GSVIVT01026993001	2264	693	77.42	9.45	40.82	107.89	L (14.1%), S (10.4%), I (6.9%)	0.306
VvFRO4	GSVIVT01028873001	3617	1178	130.07	9.41	37.52	107.21	L (12.7%), S (9.8%), A (8.2%)	0.475
VvFRO5	GSVIVT01028874001	2184	727	81.90	9.02	41.68	104.06	L (12.8%), S (10.0%), V (7.0%)	0.295
VvFRO6	GSVIVT01007662001	2342	703	79.79	9.34	47.48	106.09	L (12.5%), S (8.5%), I (8.2%)	0.290
OsFRO1	LOC_Os04g36720.1	1440	525	65.7481	9.34	48.28	110.32	L (11.8%), S (11.6%), A (9.1%)	0.252
OsFRO7	LOC_Os04g48930.1	2277	759	48.4685	9.72	57.52	95.07	L (13.8%), V (10.3%), A(10.1%)	0.060
ScFRE1	YLR214W(FRE1)	2061	686	78.877	9.49	52.16	91.77	L (9.3%), S (9.1%), V (7.6%)	0.448
ScFRE2	YKL220C (FRE2)	2136	711	80.0722	9.21	30.33	94.21	L (10.0%), A (7.9%), S (7.0%)	0.129
ScFRE3	YOR381W(FRE3)	2136	711	80.5892	6.66	35.83	94.75	L (10.3%), A (7.6), G (7.0%)	0.152
ScFRE4	YNR060W(FRE4)	2160	719	82.0153	9.26	30.27	94.02	L (10.4%), A (8.1%), I (7.0%)	0.044
ScFRE5	YOR384W(FRE5)	2085	694	80.2923	8.83	37.32	96.37	L (10.8%), I (7.2%), K (6.9%)	0.025
ScFRE6	YLL051C (FRE6)	2139	712	81.9892	9.06	33.75	103.65	L (11.7%), I (9.4%), S (8.7%)	0.153
ScFRE7	YOL152W(FRE7)	1863	620	70.9053	8.86	42.47	97.60	L (9.5%), I (9.0%), S (8.7%)	0.102
ScFRE8	YGL160W(FRE8)	1713	570	65.8400	8.88	43.99	102.46	I (10.0%), S (9.3%), L (12.5%)	-0.040
HsNOX1	ENSP00000362057	1695	564	64.8710	8.79	39.69	89.70	L (10.1%), S (8.0%), F (7.4%)	0.022
HsNOX2	ENSP00000367851	1713	570	65.3359	8.90	34.69	93.72	V (7.4%), L (10.0%), F (6.8%)	0.049
HsNOX3	ENSP00000159060	1707	568	64.9349	8.28	41.18	92.39	L (10.9%), A (7.7%), S (7.2%)	0.089
HsNOX4	ENSP00000263317	1656	578	64.1022	8.94	45.56	89.53	L (12.3%), S (7.8%), F(6.7%)	-0.081
HsNOX5	ENSP00000260364	1665	554	64.1022	8.94	45.56	89.53	L (12.3%), S (7.8%), F (6.7%)	-0.081
HsDUOX1	ENSP00000317997	4656	1551	177.235	8.12	47.83	88.56	L (11.6%), R(7.9%), S (7.5)	-0.190
HsDUOX2	ENSP00000267837	4670	1548	175.364	8.02	46.85	89.68	L (11.8%), G (7.0%), S (6.8%)	-0.148
CvFRO1	estExt_fgenesh3_pg	3364	1123	123.931	9.69	48.36	88.76	A (11.6%), L (10.9%), G (8.4%)	0.073
CrFRO2	Cre03.g188400.t1.1	1863	620	67.6035	6.54	40.95	90.37	A (12.7%), L (9.5%)，G (8.1%)	0.102
CrFRO3	Cre04.g227400.t1.2	3264	1087	114.566	9.85	44.41	88.39	A (15.3%), L (10.2%), （9.1%）	0.215
CsFRO1	estExt_Genemark1	2322	773	85.2232	7.69	43.80	106.64	L (15.5%), A (10.0%), G (8.2%)	0.290
VcFRO1	Vocar20008902m	2994	998	107.129	9.95	48.36	91.57	L (12.6%), S (11.3%), A (11.2%)	0.129
PpFRO1	Pp1s14_322V6.1	2157	718	80.8249	9.28	41.18	111.45	L (11.8%), I (8.2%), V (7.9%)	0.384
PpFRO2	Pp1s39_125V6.1	2286	761	85.1266	6.71	38.11	102.48	L (12.9%), S (8.5%), V (7.9%)	0.229
PpFRO3	Pp1s54_173V6.1	2304	767	85.6383	9.03	42.48	94.05	V (8.6%), A (8.1%), L (9.1%)	0.163
PpFRO4	Pp1s663_3V6.1	2268	755	84.7296	8.54	39.70	103.02	L (12.2%), S (8.6%), V (7.9%)	0.302
PpFRO5	Pp1s90_3V6.	2283	760	84.5003	8.76	38.48	100.26	L (11.6%), V (9.6%), G (8.0%)	0.274
PaFRO1	MA_76199g0010	2328	775	87.8788	9.35	38.05	103.59	L (12.4%), S (9.4%) V (7.5%),	0.157

Table S2. **Abbreviations:** MW, molecular weight; Pl, protein length; pIs, isoelectric point; Ii, index instability; Ai, aliphatic index; GRAVY, grand average of hydropathicity; (A, Ala; P, Pro; S, Ser; G, Gly; L, Leu:N, ASN; T,Thr).The data source of each specie is list in table 2, whereas the information about protein was taken from ExPASy (http://web.expasy.org/protparam/)

Table S3. Motif sequences identified by MEME tools

Motif	Sites	Width	E-value	Multilevel consensus sequence
1	43	29	6.7e-678	YNPTSFIFVNCPSISKLQWHPFTVTSSPN
2	43	27	1.9e-557	RYENLVMVAGGIGITPFISIIRDIIYR
3	44	19	1.1e-494	IRRKYFELFYYTHHLYIVF
4	44	21	2.6e-456	LIDIPFEHSIKYHIWLGHMMM
5	41	27	9.3e-452	DVGVLVCGPEKMQEEVAKECRSHNADN
6	38	33	8.5e-525	FCMVLPGIFLFMIDRFLRFCQSRRKVDIVSARC
7	38	29	4.7e-462	EWDRTGVANLAGEIALVAGLVMWVTSLPP
8	50	15	6.8e-279	EVCVEGPYGPESDYF
9	37	15	1.4e-246	GNICMAFLFFPVARG
10	41	21	5.0e-277	DDDKMSVVIKCQGDWTQKLYD
11	38	24	3.0e-240	VHLICAVKKSDDLCMLDLIDPQSI
12	44	21	4.4e-232	SQLNLQIEAYVTREEEPDTED
13	20	29	1.5e-225	AIISSSFVMFMLIIGIITRYYIYPIDHNT
14	33	21	8.5e-210	FTAHGLCYIIYWAMTNQIQEM
15	42	15	1.4e-207	PCDTVELNFSKPPGL
16	41	15	2.2e-181	TNVHYGCRPNFKEIF
17	33	29	5.3e-301	RPVLVDGPLGIVSAAEFLFIMMFVALLVW
18	33	29	2.0e-246	TYFGYQGTNFLVYSFPMMFIAVLGCVYLH
19	30	21	1.7e-151	LKFLMMVIFLGWIMVWIMMPT
20	14	41	3.6e-154	YPYSYRSAWNMLLMCVCIAITASAAFLWNKKQNTKEDKQIQ

Table-S4: Genomic Information of FRO genes

Gene Name	Translation ID	Sequence Position	Full length Chr	Chr	Orientation
VvFRO1	GSVIVT01023105001	22531017-22539454	22702307	12	Forward
VvFRO2	GSVIVT01026991001	18754824-18759433	20304914	15	Reverse
VvFRO3	GSVIVT01026993001	18731174-18734997	20304914	15	Reverse
VvFRO4	GSVIVT01028873001	17920090-17930273	22053297	16	Reverse
VvFRO5	GSVIVT01028874001	17911251-17919834	22053297	16	Reverse
VvFRO6	GSVIVT01007662001	10745402-10748530	17126926	17	Forward
PaFRO1	MA_76199g0010	18853- 25876	33200	18	Forward
OsFRO1	LOC_Os04g36720.1	22182599-22186943	35502694	4	Forward
OsFRO2	LOC_Os04g48930.1	29178862-29181665	35502694	4	Reverse
ZmFRO1	GRMZM2G089291_T01	88970062-88974958	307041717	1	Reverse
ZmFRO2	GRMZM2G068557_T01	46785444-46790374	244442276	2	Reverse
BdFRO1	Bradi5g11147.1	14832325-14837374	28563423	5	Reverse
BdFRO2	Bradi5g19150.1	22305120-22308896	28563423	5	Reverse
CrFRO1	Cre03.g188300.t1.1	5747176-5751750	9200000	3	Forward
CrFRO2	Cre03.g188400.t1.1	5753406-5758534	9200000	3	Forward
CrFRO3	Cre04.g227400.t1.2	3355079-3363615	4000000	4	Forward
SmFRO1	e_gw1.0.1110.1	3570486-3573210	6,951,972	Sca_0	Forward
SmFRO2	e_gw1.39.66.1	1077630-1079463	1,708,524	Sca_39	Forward
SmFRO3	e_gw1.45.93.1	1271397-1273747	1,506,775	Sca_45	Reverse
SmFRO4	estExt_fgenesh1_pm	237856 -240422	**266977**	Sca_7	Reverse
SmFRO5	estExt_fgenesh2_pg	1890551-1893253	3,093,912	Sca_10	Reverse
SmFRO6	estExt_fgenesh2_pg	1191056-1194130	1,266,795	Sca_59	Forward
SmFRO7	estExt_Genewise1.	1893670-1896353	3,093,912	Sca_10:	Reverse
SmFRO8	fgenesh1_pm	59562-61662	235874	Sca_101	Forward
SmFRO9	gw1.9.156.1	889190-891684	3150000	Sca_9	Forward
VcFRO1	Vocar20008902m	13806643-13818846	14100000	Sca_1	Reverse
CsFRO1	estExt_Genemark1.C_30426	2251468-2257154	3000000	Sca_3	Forward
CvFRO1	estExt_fgenesh3_pg.C_60163	1277608-1284529	2000000	Sca_6	Reverse

Table-S4: Genomic Information of genes Cont……

Gene Name	Translation ID	Sequence Position	Full length Chr	Chr	Orientation
ScFRE1	YLR214W (FRE1)	568567-570627	1078177	12	Forward
ScFRE2	YKL220C (FRE2)	9091-11226	666816	11	Reverse
ScFRE3	YOR381W (FRE3)	1055545-1057680	1091291	15	Forward
ScFRE4	YNR060W (FRE4)	739951-742110	784333	14	Forward
ScFRE5	YOR384W (FRE5)	1061564-1063648	1091291	15	Forward
ScFRE6	YLL051C (FRE6)	37333-39471	1078177	12	Reverse
ScFRE7	YOL152W (FRE7)	40748-42610	1091291	15	Forward
ScFRE8	YGL160W (FRE8)	239347-241407	1078177	12	Forward
AtFRO1	AT1G01590.1	214150 - 217734	30427671	1	Forward
AtFRO2	AT1G01580.1	209208 - 213080	30427671	1	Forward
AtFRO3	AT1G23020.1	209208 - 213080	30427671	1	Forward
AtFRO4	AT5G23980.1	8097978 - 8101330	26975502	5	Reverse
AtFRO5	AT5G23990.1	8105565 - 8108590	26975502	5	Reverse
AtFRO6	AT5G49730.1	20201037 - 20204595	26975502	5	Reverse
AtFRO7	AT5G49740.1	20205302 - 20208777	26975502	5	Reverse
AtFRO8	AT5G50160.1	20415764 - 20418847	26975502	5	Forward
PtFRO1	Potri.001G079000.1	6257724 - 6264650	48367220	1	Reverse
PtFRO2	Potri.004G079100.1	6550267 - 6553701	23188140	4	Reverse
PtFRO3	Potri.004G079200.1	6557306 - 6561136	23188140	4	Reverse
PtFRO4	Potri.012G084800.1	11220313 - 11224179	14929429	12	Forward
PtFRO5	Potri.014G088000.1	6946787 - 6951800	17716633	14	Reverse
PtFRO6	Potri.015G083200.1	10742214 - 10745893	15134944	15	Forward
PtFRO7	Potri.017G142700.1	15005978 - 15009498	14661173	17	Reverse
PtFRO8	Potri.017G142800.1	15009803 - 15013806	14661173	17	Reverse
PpFRO1	Pp1s14_322V6.1	13113522 - 13118092	19500000	6	Forward
PpFRO2	Pp1s39_125V6.1	480603 - 485383	17500000	11	Reverse
PpFRO3	Pp1s54_173V6.1	1325642 - 1330056	5300000	27	Reverse
PpFRO4	Pp1s663_3V6.1	7326760 - 7332442	16500000	16	Forward
PpFRO5	Pp1s90_3V6.1	22657 - 27233	17800000	9	Reverse

Table S5. The cis-elements regulatory elements in the promoter region of OsFRO1& OsFRO7 gene

***Cis*-elements**	**Core sequences**	**Functions of the cis-elements**	**Position Strand**
		**LOC_Os04g48930 (OsFRO1)**	
ABRE	AGTACGTGGC	cis-acting element involved in the abscisic acid responsiveness	1255+
GC-motif	AGCGCGGCC	Unknown	1200+
P-box	CCTTTTG	gibberellin-responsive element	1375+
Skn-1-motif	GTCAT	cis-acting regulatory element required for endosperm expression	720+
Sp1	GGGCGG	light responsive element	358-
Sp1	GGGCGG	light responsive element	1141+
Motif-IIb	CCGCCGCGCT	abscisic acid responsive element	1200-
TATA -box	TACAAAA	core promoter element around -30 of transcription start	716-
Plant-Ap-2-Like	CGCGCCGG	Unknown	198+
		**LOC_Os04g36720 (OsFRO7)**	
P-box	CCTTTTG	gibberellin-responsive element	86-
Skn-1-motif	GTCAT	cis-acting regulatory element required for endosperm expression	425+
TATA -box	TACAAAA	core promoter element around -30 of transcription start	384_
GC-motif	GCCGCGCG	Unknown	1200+

Table. S6: The detailed list of primers used for expression analysis

Gene	Primer (5'→3')	Product size	Exon No
**Primers used for semi-quantitative RT-PCR analysis.**			
**Gene**	**Primer (5'→3')**	**Product size**	**Exon No**
OsFRO1 Forward	ATTGTGACAGGCATAGAACTCG	333 bp	1
OsFRO1 Reverse	GACTGGAAAGAACAGGAACG		
OsFRO7 Forward	GCCTGCTAATGTGGGTGAC	273 bp	1
OsFRO7 Reverse	GGAGACTTGCTGGCTTTGA		
OsActin1 Forward	CAGCACATTCCAGCAGATGT	198 bp	2
OsActin1 Reverse	TAGGCCGGTTGAAAACTTTG		
**Primers used for real-time qRT-PCR analysis**			
**Gene**	**Primer (5'→3')**	**Product size**	**Exon No**
OsFRO1 Forward	TCCTGTTCTTTCCAGTCGC	284 bp	1
OsFRO1 Reverse	GTTCTTGTCCATTTCAGCATCT		
OsFRO7 Forward	CTGGCATTTCACGTTGGC	271 bp	1
OsFRO7 Reverse	CGGAGACTTGCTGGCTTTG		
OsActin1 Forward	GTGGTCGCCCCTCCTGAAAG	198 bp	2
OsActin1 Reverse	GGCTTAGCATTCTTGGGTCCG		
